# Critical assessment of uncertainty in economic evaluations on influenza vaccines for the elderly population in Spain

**DOI:** 10.1186/s12879-025-10442-3

**Published:** 2025-02-01

**Authors:** Raúl Ortiz-de-Lejarazu Leonardo, Javier Díez Domingo, Ángel Gil de Miguel, Federico Martinón Torres, Esther Redondo Margüello, Juan Luis López-Belmonte Claver, Paloma I. Palomo-Jiménez, J. Manel Farré Avellà, José María Abellán Perpiñán

**Affiliations:** 1 Centro Nacional de Gripe de Valladolid, GISRS (Global Influenza Surveillance and Response System), Valladolid, Spain; 2 Fundación para el Fomento de la Investigación Sanitaria y Biomédica de la Comunitat Valenciana (FISABIO), Valencia, Spain; 3 Preventive and Public Health Department, Rey Juan Carlos University, Madrid, Spain; 4 Translational Paediatrics and Infectious Diseases Section, Paediatrics Department, Hospital Clínico Universitario de Santiago de Compostela, Santiago de Compostela, Spain; 5 Vaccines, Infections and Pediatrics Research Group (GENVIP), Healthcare Research Institute of Santiago de Compostela, Santiago de Compostela, 15706 Spain; 6 International Healthcare Centre of Ayuntamiento de Madrid, Madrid, 28006 Spain; 7 CIBER of Respiratory Diseases (CIBERES), Instituto de Salud Carlos III, Madrid, 28029 Spain; 8 Sanofi, Market Access, Madrid, Spain; 9 Sanofi, Medical Affairs, Barcelona, Spain; 10 Applied Economics Department, Economics & Enterprise Faculty, University of Murcia, Murcia, Spain

**Keywords:** Cost-effectiveness analysis, Influenza vaccination, Uncertainty analysis, TRUST Tool, Quality of evidence

## Abstract

**Background:**

Influenza is a seasonal infection with a huge impact on morbidity and mortality in older adults, for whom vaccination is recommended. New influenza vaccines for this population have been introduced in Spain in the past 5 years, and a number of cost-effectiveness analyses (CEA) have been published to aid healthcare decision-making. The objective of this study was to assess possible sources of uncertainty in the CEAs of influenza vaccines for the older adults in Spain.

**Methods:**

A systematic review was performed to identify Spanish CEAs published since 2016. Potential sources of structural, methodologic and parametric uncertainty in CEA results were systematically analysed using the TRansparent Uncertainty ASsessmenT (TRUST) Tool, quality assessment checklists, and the WHO guidance on economic evaluations of influenza vaccine strategies. The primary sources of efficacy/effectiveness were analysed in depth to ascertain whether they could support the conclusions of the respective CEAs.

**Results:**

Seven CEAs were included. Overall, they were designed and performed in accordance with the applicable guidelines; however, some critical sources of uncertainty were detected, mainly: (1) the choice and use of efficacy/effectiveness data (real-world single season studies, meta-analyses including studies with high risk of bias and/or high heterogeneity with biased interpretation); (2) use of fewer than 5 seasons to estimate influenza burden; (3) generalized use of influenza-like illness data to estimate effectiveness and burden, among others.

**Conclusions:**

Seemingly well-designed studies may conceal important sources of uncertainty that affect the results. This must be taken into account when interpreting results to support decision-making.

**Supplementary Information:**

The online version contains supplementary material available at 10.1186/s12879-025-10442-3.

## Introduction

 Influenza is an annual viral illness that causes seasonal epidemics [1]. In Spain, it costs up to €145–1,000 million annually [2]. Hospitalizations, mainly in high-risk groups, are the key cost-driver [2]. The population over 65 is especially susceptible to severe complications [3, 4], so the WHO recommends seasonal immunization in this group [5], and the Spanish national healthcare system (NHS) [6, 7] has implemented a vaccination program to prevent severe infections, hospitalizations, and deaths. Standard dose quadrivalent (QIV) vaccines are effective in preventing influenza and its complications [8]. However, various studies have reported that these vaccines are less effective in elderly adults due to immunosenescence [9]. For this reason, adjuvanted QIV (aQIV) and high-dose QIV (HD-QIV) vaccines have been developed to specifically address immunosenescence issues, but are more expensive. Cell-cultured (QIVc) and, more recently, recombinant vaccines (QIVr) aim to overcome viral mutation occurring in egg-based manufacturing process.

Spain has a decentralized healthcare system in which each autonomous region can decide the type of vaccine to be used in target influenza vaccination populations. However, due to budget constraints, each region must carefully evaluate the cost-effectiveness of all available options in order to balance resource allocation with clinical results [10]. Therefore, critical reading and interpretation of available economic evaluations is paramount in the decision-making process.

Cost-effectiveness analyses (CEA) measure the incremental health gains and costs of interventions in order to assess the efficiency of funding a new healthcare intervention with respect to other established ones in a specific setting. Differences in model type, methods, and parameter values introduce uncertainty, and policymakers require thorough description and quantification to understand the strengths and limitations of the analysis [11].

For this purpose, we systematically reviewed and analysed the CEAs of influenza vaccines for the older adults (≥ 60 years) published in Spain to: (1) assess whether these evaluations were carried out following best practices in economic evaluation of influenza vaccines, (2) identify possible sources of uncertainty that may affect the results and should be considered in decision making.

## Methods

**Fig. 1:**
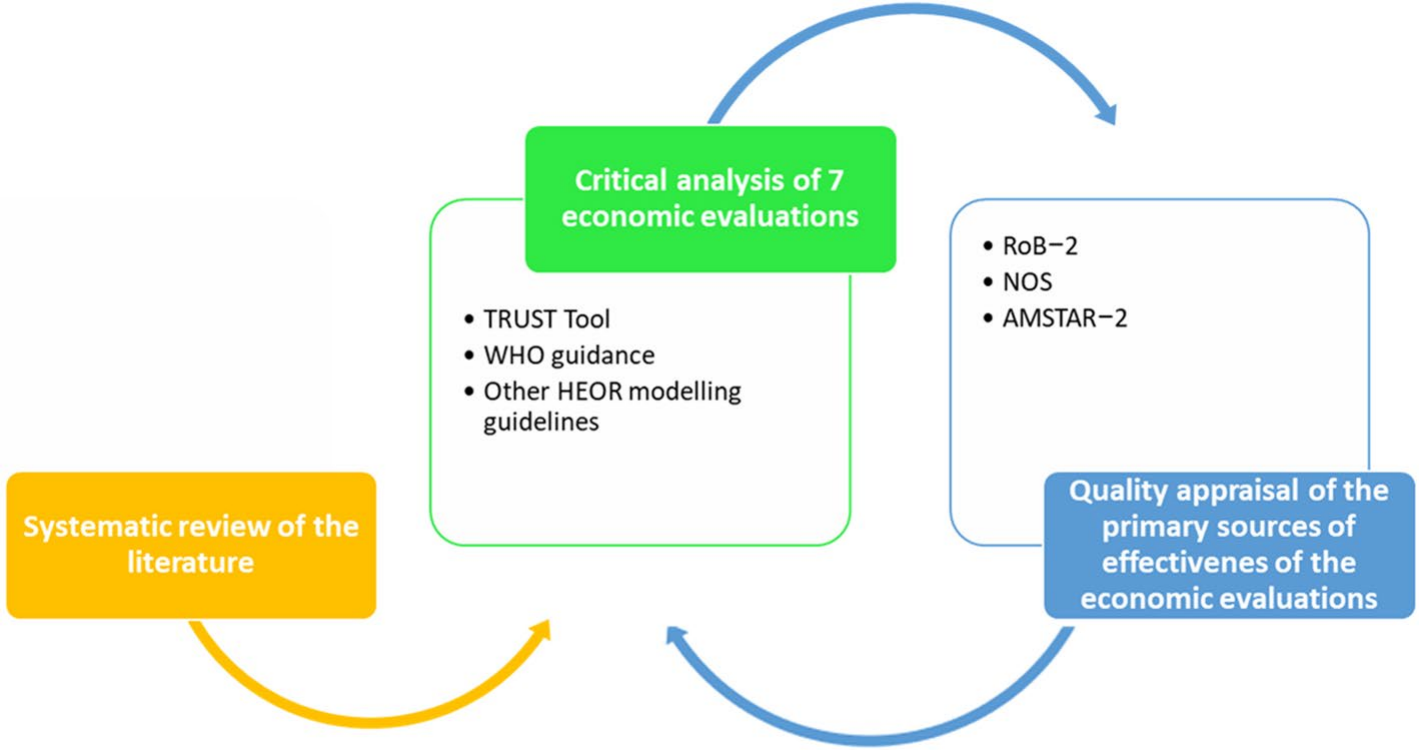
Study flowchart AMSTAR-2: A Measurement Tool to Assess Systematic Reviews-2; HEOR: Health-Economics and Outcomes Research; TRUST: TRansparent Uncertainty ASsessmenT; WHO: World Health Organization; RoB 2: Risk of bias 2; NOS: Newcastle-Ottawa Scale

To fulfil our objective, we first collected the available studies assessing the cost-effectiveness of influenza vaccines in Spain, then we have critically analyzed different uncertainty sources (i.e., parametric, structural and methodological) surrounding each of the identified studies using an influenza-adapted TRUST Tool. When analysing the parametric uncertainty of each CEA we have also retrieved all efficacy/effectiveness sources and appraised their methodological quality by using the appropriate validated checklist (RoB2, AMSTAR-2, or NOS). The study flowchart is shown in Fig. 1.

### Systematic review

We performed a systematic review of the literature reporting complete economic evaluations (CEE), i.e., cost-effectiveness, cost-utility analyses, and/or cost-benefit analyses of influenza vaccines in the population aged > 60 years in Spain, following the recommendations of the Cochrane Handbook for Systematic Review of Interventions [12]. PubMed (https://pubmed.ncbi.nlm.nih.gov/ ), Scopus (https://www.scopus.com/home.uri ), Web of Sciente (WoS), and the Biblioteca Virtual de Salud (https://bvsalud.isciii.es/) were searched for studies published since 2016 in Spanish or English. The search was closed on November 7th, 2022. One reviewer screened the references, with the others being available for resolving doubts. The complete definition of the review question, search terms and strategy, and the full selection criteria are detailed in Supplementary Material 1.

### Critical analysis of the economic evaluations

Each study was critically reviewed by three independent investigators to verify their the compliance with the WHO “Guidance on the economic evaluation of influenza vaccination” [13] and the TRansparent Uncertainty ASsessmenT (TRUST) Tool [14] was used to identify possible sources of uncertainty in CEEs. A consensus of the most relevant sources of uncertainty in each study was reached through discussion among the three investigators.

As the TRUST Tool has not been developed specifically for influenza vaccine models, we first created a theoretical framework for the tool by collecting all theoretical sources for TRUST cited by Grimm et al. 2020 [14] and the recommendations from the WHO guidance [13] and merging them with unique definitions to give all investigators a common standard for their analysis.

It is also important to correctly define the terminology used to describe the types of uncertainty assessed in this article, as this is sometimes confusing in the literature:

#### Model or structural uncertainty

Model uncertainty arises from the choice of the model (e.g., dynamic or static), its structure, and how health states are connected. It also analyses whether the model correctly assesses the main objective [11, 15–17]. Issues such as transparency, biases in modelling choices, and uncertainties around key structural assumptions (e.g., time horizons or exclusion of health states) may need further exploration, e.g., through sensitivity analyses [14].

#### Methodological uncertainty

Methodological uncertainty arises from the various economic evaluation methods used to estimate use of resources and the health outcomes of the assessed interventions. It can arise from the chosen perspective, time horizon, analytic technique (CEA, CEU), discount rate, etc [11]. Methodologic inconsistencies or deviations from standardized procedures warrant scenario analyses or the incorporation of expert opinion to buffer their impact on results [14].

#### Parameter uncertainty

This uncertainty arises from the specific data used to populate the model, and therefore involves efficacy and/or effectiveness data, unit costs, resource use/costs, epidemiological burden of the disease, etc [17]. Parameter uncertainty is a second-order uncertainty and relates to the fact that the probabilities that govern outcomes, being estimated quantities, are themselves uncertain [15]. Parameter uncertainty also encompasses imprecision, such as wide confidence intervals or immature data, and may reflect data biases or indirectness. To address this, probabilistic sensitivity analysis (PSA) should be used to quantify the effects of parameter uncertainty [14].

#### Management of uncertainty

Managing uncertainty in CEAs is crucial [17]. Sensitivity and scenario analyses, including deterministic sensitivity analysis (DSA) and probabilistic sensitivity analysis (PSA), are of great importance [17]. In DSA, one parameter at a time is varied within its realistic limits, often from 95% confidence intervals. This clarifies the impact of a specific variable on cost-effectiveness. PSA, a multivariate analysis simultaneously varies all variables based on probability distributions, thereby creating cost-effectiveness planes and acceptability curves relative to a defined willingness-to-pay threshold for each country.

### Assessment of the quality of the primary effectiveness sources

The data sources for vaccine effectiveness in each CEE were identified, retrieved, and analysed as follows: (1) three investigators independently evaluated the global quality of each publication by applying one of the most commonly used [18] validated quality appraisal checklists, according to the type of study (Table 1); (2) Each source and the conclusions of the previous technical verification were then critically reviewed by a clinical expert, who examined aspects specific to influenza that are not evaluated in quality checklists, such as case definition, number of seasons included, and population subgroups. (3) All studies, analyses, and conclusions were shared and discussed in two investigator meetings held in 2023, in which the participants agreed on their conclusions and decided whether the effectiveness data were sourced from the best studies available at the time of model development, and whether original data were correctly used in the model.

**Table 1:** Quality assessment checklist used according to the type of study

Type of study	Checklist **[**18**]**
Randomized controlled trial	RoB 2 [19]
Observational study	NOS [20]
Systematic review with meta-analysis	AMSTAR-2 [21]

*AMSTAR-2 *A MeaSurement Tool to Assess systematic Reviews 2,* NOS *Newcastle-Ottawa Score,* RoB 2 *Risk of Bias 2

## Results

### Analysed studies

The literature search yielded 163 primary studies, of which 94 were unique references; 85 were excluded by title/abstract (mainly because they were centred in a different population or used other types of economic evaluations) and 9 were included for full-text reading. Six were finally included. However, an additional study that met the selection criteria was published after closing the search and was added manually. The Preferred Reporting Items for Systematic Review and Meta-Analysis (PRISMA) flowchart for the selection process and the list of excluded studies and the motives for exclusion are presented in Supplementary Material 2.

The main characteristics of the included studies are detailed in Table 2.

**Table 2:** Main characteristics of the economic evaluations analysed

Reference	Type of economic evaluation	Type of model	Evaluated vaccine type (unit price) Reference year	Age groups	Time horizon	Perspective	Results and conclusions	Sponsored by
**Garcia et al. 2016** [31]	Cost-utility	Static decision tree	QIV (€9.50) vs. TIV (€7.00) Year 2014	0–4, 5–17, 18–49, 50–64, 65–69, 70–74, 75–79, 80–84 and 85+	100 years	NHS and societal	Compared to TIVs, QIV reduced more influenza cases and influenza-related complications and deaths during B-mismatch periods. ICER was €8,748/QALY. DSA showed that mismatch with the B lineage included in the TIV was the main driver for ICER. *Authors conclude that replacing TIVs with QIV in Spain could be a cost-effective healthcare intervention.*	GlaxoSmithKline
**Crepéy et al. 2020** [29]	Cost-utility and budgetary impact	Dynamic transition model	QIV (€9.50) vs. TIV (€7.15) Year 2017	< 65 high risk and 65+	1 year	NHS and societal	Replacing TIV with QIV may have prevented 138,707 influenza B cases per season and avoided 10,748 outpatient visits, 3,179 hospitalizations, and 192 deaths. This could save €532,768 in outpatient visits, €13 million in hospitalizations, and €3 million in influenza-related deaths per year. An additional €5 million associated with productivity loss could be saved per year from the societal perspective. The ICER was €1,527/QALY. *Thus*,* authors consider QIV to be cost-effective compared to TIV.*	Sanofi Pasteur
**Ruiz-Aragón et al. 2020** [32]	Cost-utility	Static decision tree	eQIV (€6.00) vs. cQIV (€7.50) Year not stated	9–17; 18–60; 60–64	1 year + long-term consequences (indirect costs)	NHS and Societal	Using QIVc instead of QIVe was associated with 16,221 fewer symptomatic cases, 4,522 fewer primary care visits, 1,015 fewer emergency room visits, and 88 fewer hospitalizations. From a societal perspective, QIVc was more effective and less expensive compared to QIVe, leading to a cost saving of €3.4 million. From a public payer perspective, the ICER for QIVc vs. QIVe was €12,852/QALY. *Authors conclude that QIVc offers a cost-effective alternative to QIVe and should be considered for people aged 9–64 at high-risk of influenza complications*.	Seqirus
**Redondo et al. 2021 **[35]	Cost-utility	Static decision tree	HD-QIV (€32.00) vs. aQIV (€8.00) Year 2020	65+	1 season	NHS	Switching from aTIV strategy to HD-QIV would prevent 36,476 cases of influenza, 5,143 visits to GP, 1,054 visits to the ED, 9,193 hospitalizations due to influenza or pneumonia, and 357 deaths due to influenza. This would result in a gain of 3,514 life years and 3,167 QALYs. Healthcare costs increased by €78,874,301, leading to an incremental cost-effectiveness ratio of €24,353/QALY. *Authors conclude that HD-QIV would be cost-effective compared to aTIV in the population under study.*	Sanofi Pasteur
**Ruiz-Aragón et al. 2022 **[34]	Cost effectiveness and Cost utility	Static decision tree	aQIV (€13.00) vs. HD-QIV (€25.00) Year 2021	65+	1 year + long-term consequences (indirect costs)	NHS and Societal	aQIV vs. HD-QIV yielded reductions of 5405 symptomatic cases, 760 primary care visits, 171 emergency room visits, 442 hospitalizations, and 26 deaths in Spain each year. LYs and QALYs increases by 260 and 206, respectively, each year. Savings from a direct medical payer perspective are €63.6 million, driven by the lower aQIV vaccine price and a minor advantage in effectiveness. From a societal perspective, savings increase to €64.2 million. When vaccine prices are assumed equal, aQIV remains dominant compared to HD-QIV. Potential savings are estimated at over €61 million in vaccine costs alone. *Authors affirm that aQIV is a highly cost-effective alternative to HD-QIV for people aged 65 + in Spain.*	Seqirus
**Fochesato et al.**,** 2022 **[30]	Cost-utility	Dynamic transition model	aQIV (€13.00) vs. SD-QIV (€9.50) Year 2021	6–23 months; 2–6 years; 7–17 years; 18–64 years; 65+	1 season + long-term consequences (indirect costs)	NHS and Societal	Replacing QIVe with aQIV would prevent, on average, 43,664 complicated cases, 1111 hospitalizations, and 569 deaths (rVE = 34.6%) or 19,104 cases of influenza complications, 486 hospitalizations, and 252 deaths (rVE = 13.9%). When the rVE of aQIV vs. QIVe is 34.6%, ICER was €2240/QALY from the payer perspective; from the societal perspective, aQIV was cost saving compared with QIVe. With an rVE of 13.9%, ICER was €6694/QALY and €3936/QALY from the payer and societal perspective, respectively. *According to authors*,* aQIV in the Spanish older adults is cost effective for the Spanish NHS compared to QIVe.*	Seqirus
**Ruiz Aragón et al. 2023** [33]	Cost-utility	Static decision tree	QIVr (€25.00) vs. aQIV (€13.00) Year 2021	65+	1 season + long term consequences (indirect costs)	Societal	Based on current tender prices and assuming QIVr/aQIV rVE of 10.7%, the ICER for QIVr was €101,612.41/QALY. Authors also estimated that to meet the Spanish WTP threshold of €25,000/QALY, rVE for QIVr vs. aQIV should be 34.1%. In their PSA, 99.7% of simulations for QIVr were higher than the WTP curve. *QIVr is not currently a cost-effective influenza vaccine option relative to aQIV for elderly individuals in Spain.*	Seqirus

*aQIV* Adjuvated-Quadrivalent Influenza Vaccine, *aTIV* Adjuvated Trivalent Influenza Vaccine, *DSA* Deterministic Sensitivity Analysis, *ED* Emergency Department, *GP* General Practice, *HD-QIV* High-Dose Quadrivalent Influenza Vaccine, *NHS* National health service, *PSA* Probabilistic sensitivity analysis, *rVE* relative Vaccine Effectiveness, *QIV* Quadrivalent Influenza Vaccine, *QIVe* egg-based Quadrivalent Influenza Vaccine, *QIVc* cell-based Quadrivalent Influenza Vaccine, *QIVr* Recombinant Quadrivalent Influenza Vaccine *SD-QIV* Standard-Dose Quadrivalent Influenza Vaccine, *TIV* Trivalent Influenza Vaccine, *WTP* Willingness to pay

The quality appraisal of the analysed studies according to validated checklists is reported in the Supplementary Material 3. The quality of the sources used by each study was highly heterogeneous, ranging from high quality RCTs [22] to critically-low-quality systematic reviews with meta-analysis [23–26]. Some sources could not be evaluated because they were not available or did not offer sufficient detail [27]. However, the general methodologic quality assessment did not capture all relevant aspects concerning influenza vaccines, for which a thorough analysis of these aspects was carried out when applying the TRUST tool.

### Description of sources of uncertainty in the included CEA

As described, the TRUST [14] tool and WHO guidance [13, 28] were used to assess potential uncertainty in several domains. Table 3 describes the analysis of sources of potential parameter uncertainty and Table 4 describes the assessment of methodological uncertainty. The detailed analysis by study is reported in Supplementary Material 4. Table 5 analyses the primary sources of effectiveness data used in each economic study.

**Table 3:** TRUST tool analysis of sources of parametric uncertainty

	Burden of illness (< 5 seasons)	Characteristics of the source of effectiveness data^a^		Baseline utility and disutility values	Resource use & costs data	Other	Sensitivity analyses
		Design of sources					
**García et al. 2016 **[31]	GP or ED 1 year; hospitalization and death 6 years	• QIV VE: Tricco et al. 2013, many included studies have uncertain results with 95%CIs crossing RR = 1; heterogeneity is moderate	GP or ED cases: ILI. Hospitalization and death: Lab-confirmed influenza and ILI	NRUD	Includes children, but productivity loss for working parents is not calculated	NRUD	Parameters tested in DSA and their intervals were not listed. Authors state that the parameters most affecting the ICER of QIV were: the circulation of type A influenza, and the potential B lineage mismatch; but tornado plot is not provided. PSA was performed but probability distributions were not declared, results are represented as acceptability curve
**Crépey et al. 2020 **[29]	NRUD	• TIV VE and additional B strain VE: Meta-analysis Diaz Granados et al. 2012 and CDC unpublished data at the time of the study.	NRUD	NRUD	Includes children but productivity loss for working parents is not calculated. Resource costs derived from eSalud^b^, primary data sources and data processing not explained	NRUD	DSA variation intervals and PSA probability distributions were not informed. PSA is not represented as an acceptability curve
**Ruiz Aragón et al. 2020 **[32]	3 seasons (2014/15- 2017/18)	• QIVc and QIVe A/H1N1 and B: Belongia et al. 2016 meta-analysis: only includes test-negative design studies • QIVc A/H3N2, from Boikos et al. 2019 from single season unpublished observational study.	QIVc: ILI	Model age groups do not correspond to the age groups defined in the data source; method of mapping original utility values on the model groups should have been explained.	Resource cost derived from official bulletins of two regions, reason for choice and management of costs not given	Vaccine coverage from data-on-file provided by the intervention manufacturer, while vaccination coverage is publicly available from official Spanish sources.	A one-way deterministic sensitivity analysis (DSA) was conducted where model parameters were varied by ± 10%, which is not the recommended method in applicable guidelines. The tornado plot representing the variables having the highest impact is presented. Also, PSA was carried out and represented as a cost-effectiveness plane (scatterplot). .
**Redondo et al. 2021 **[35]	NRUD	• rVE aTIV vs. SD-TIV: Puig Barberá et al. 2016 RWE for 1 season	Lab-confirmed influenza; ILI for HD-QIV vs. SD-QIV protection against hospitalization	NRUD	NRUD	NRUD	A scenario analysis is provided testing a broader definition of influenza complication for hospitalizations and 0% rVE for aTIV vs. SD-TIV. All upper and lower bound used in the DSA are detailed in the paper, as well as probability distributions used in the PSA, making the sensitivity analysis highly transparent. Sensitivity analyses are presented for both base case and alternative scenario. DSA is presented by tornado diagram, and PSA by acceptability curve, while the cost-effectiveness plane was not represented
**Ruiz Aragón et al. 2022 **[34]	3 seasons (2017/18–2019/20)	• High-heterogeneity meta-analysis not based on systematic search	Not specified	No explanation of method used to map disutility values from original age groups to model age groups	Resource costs derived from official bulletins of three regions, reason for choice not given. Middle cost used.	NRUD	Both PSA and DSA have been carried out; however, DSA input data and probability distributions used in the PSA have not been reported.
**Fochesato et al. 2022 **[30]	Not specified	• VE for QIVe Belongia et al. 2016 meta-analysis: only includes test-negative design studies	Alternative scenario ILI	Italian data used even though Spanish data were available	Resource cost derived from official bulletins of different regions, reason for choice and management of costs not given	Contact rates from Italy transferred to Spanish model. Data were considered transferable when at least one circulating strain was present in both countries simultaneously. However, this strain was usually H1N1 A, subtype against which all vaccines have high effectiveness for the elderly. For four seasons, circulating strains did not coincide - either H3N2 or B (or both) were present in one of the countries but absent in the other. Model calibration not presented.	An alternative scenario was estimated with rVE coming from Coleman et al. 2021. DSA and PSA are carried out for both the base case and the alternative scenario. Upper and lower bounds for sensitivity analyses are detailed. DSA is represented through tornado plots and the PSA is represented as a cost-effectiveness scatter plot.
**Ruiz Aragón et al. 2023 **[33]	3 seasons (2017/18–2019/20)	• RWE of QIVr vs. aTIV based on only 1 season	Not specified	NRUD	Resource cost derived from official bulletins of three regions, reason for choice and management of costs not given	NRUD	DSA and PSA were carried out to test the robustness of the model, which are both represented in the paper with a tornado plot and the cost-effectiveness plane, respectively. While DSA upper and lower bounds were presented, their source is not cited. Probability distribution for PSA is not presented either. Considering the uncertainty of the available effectiveness data, authors carried out a scenario analysis in which they fixed the ICER at 25,000€/QALY, which is commonly considered as the acceptability threshold in Spain, and calculated back the necessary rVE for QIVr vs. aQIV, it resulted in 34.12%, authors discuss that this efficacy has not been reached even in the RCT.

*aTIV* Adjuvated-TIV, *CDC* Centre for Disease Control, *GP* General practice, *ED* Emergency department, *HD-QIV* High-dose-QIV, *ILI* Influenza-like illness, *NRUD* No relevant uncertainties detected, *RWE* Real world evidence, *QIV* Quadrivalent inactivated vaccine, *QIVc* cell-based-QIV, *QIVe* egg-based-QIV, *QIVr* recombinant-QIV, *SD-TIV* Standard-dose-TIV, *TIV* Trivalent inactivated vaccine

^a^Quality and appropriateness of sources of effectiveness were assessed as described in Methods

^b^eSalud is a Spanish online repository of costs from different sources, mainly official bulletins, and published literature

**Table 4:** TRUST tool analysis of sources of methodological uncertainty: outcome presentation and uncertainty management

	Perspective	Disaggregated outcomes (costs and benefits)	Disaggregated ICER (NHS and societal perspectives)	Scenario analysis	Deterministic sensitivity analysis		Probabilistic sensitivity analysis		Acceptability threshold
					Presentation of input data (upper/lower limits and sources)	Graphic representation (Tornado plot)	Presentation of input data (distributions)	Graph	
**García et al. 2016 **[31]	NRUD	NRUD	NRUD	NA	Not presented	No graph	Not presented	Only acceptability curve	€20,000/QALY and €30,000/QALY
**Crépey et al. 2020 **[29]	NRUD	NRUD	NRUD	NA	Not presented	Tornado plot	Distributions	No graph	€25,000/QALY
**Ruiz Aragón et al. 2020 **[32]	NRUD	NRUD	NRUD	NA	Variation based on arbitrary intervals: ±10%, does not follow ISPOR guidelines	Tornado plot	Distributions	Only C/E plane	€22,000/QALY €25,000/QALY
**Redondo et al. 2021 **[35]	No societal perspective	NRUD	NA	Alternative scenario testing a broader definition of influenza complications for hospitalizations and 0% rVE for aTIV vs. SD-TIV. Sensitivity analysis also presented	NRUD	Tornado plot	Distributions	Acceptability curve and C/E plane	€30,000/QALY
**Ruiz Aragón et al. 2022 **[34]	NRUD	NRUD	NRUD	Alternative scenario with aQIV vs. HD-QIV rVE based on Coleman et al. 2021.	Not presented	Tornado plot	Not presented	Only C/E plane	€25,000/QALY
**Fochesato et al. 2022 **[30]	NRUD	NRUD	NRUD	Alternative scenario with rVE from Coleman et al. 2021.	NRUD	Tornado plot	Distributions	Only C/E plane	€25,000/QALY
**Ruiz Aragón et al. 2023 **[33]	No NHS perspective, Societal perspective not reported	Not presented	NA	Reverse calculation: rVE for QIVr vs. aQIV calculated after pegging ICER at 25,000€/QALY	Not presented	Tornado plot	Not presented	Only C/E plane	€25,000/QALY

*C/E* Cost-effectiveness, *ICER* Incremental cost-effectiveness ratio, *NA* Not applicable, *NHS* National Health System, *NRUD* No relevant uncertainties detected, *QALY* Quality-adjusted life years

**Table 5:** Characteristics and analysis of the primary sources of effectiveness data

**Reference of the effectiveness source**	**Extracted data**	**Type of study**	**Description of the study**	**Summary of analysis with respect to use in EE**
***García et al. 2016*** [31]				
**Jefferson et al. 2010** [42]	VE of the three common influenza strains in the older adults	Cochrane SLR with MA of VE in 65+ people	Mainly non-randomized studies. Heterogeneity between studies was low (I^2^<30%). However, risk of bias was evaluated as moderate-to-high, therefore, authors deemed results to be inconclusive.	While the quality of the individual studies appears insufficient, the body of evidence may be adequate for the purpose pursued in the CEA; in fact, the value used is applied to both comparators.
**Tricco et al. 2013**[23]	VE non-matching B strain for QIV = TIV VE in complete match	SLR with MA	MA of 34 RCTs, including 49 seasons and 94,821 participants. Moderate heterogeneity (I^2^<50%) between studies; RR of various individual studies crossing the 1 line, thus yielding inconclusive MA results.	The non-matching value is proportionally applied in the CEA according to the degree of B-matching. which is averaged over 9 seasons. Thus, taken altogether the rVE estimation may be adequate.
***Crépey et al. 2020*** [29]				
**Diaz Granados et al. 2012**[60]	TIV VE and additional B strain VE for each age group	SLR with MA	MA of 30 RCTs in children and young adults, including 26 seasons and 88,468 participants. Most studies had low risk of bias; however, one sixth of them were low quality. Heterogeneity was moderate for children (I^2^=57%) and low for young adults (I^2^=14%)	This source seems adequate for younger age groups in the model
**Centre for Disease Control**		Not specified	Not specified, original source not cited and unavailable	Cannot be evaluated. However, input values for the model are transparently presented in the CEA
***Ruiz Aragón et al. 2020***[32]				
**Belongia et al. 2021** [24]	VE of QIVe for A/H1N1 and B = QIVc QIVe VE against the A/H3N2 strain	SLR with MA	MA of 56 RW studies of VE with test-negative design and lab-confirmed influenza.	Test-negative design is only one of the available designs for evaluating VE; thus, not all available evidence is included
**Boikos et al. 2019** [27]	rVE QIVe vs. QIVc against strain A/H3N2	RW study	RW single-season study (2017-18) of ILI cases	Could not be evaluated because it is an oral communication not publicly available. Other available studies could have been used; however, the reason for this source choice is not given
***Redondo et al. 2021*** [35]				
**Govaert et al. 1994** [61]	VE for SD-TIV	RCT	RCT of SD-TIV vs. no vaccination in individuals aged 60+ (*n*=1838)	Robust though rather old study; no studies have been published more recently with the same quality
**Diaz Granados et al. 2014** [22]	rVE HD-QIV vs. SD-QIV - infection	RCT	RCT of HD-TIV vs. SD-TIV in individuals aged 65+ (*n*=31,989). Two seasons (2010-11 y 2011-12)	Robust RCT. Its results were assumed to be applicable to HD-QIV vs. SD-QIV based on an immune-bridging clinical trial supported by EMA
**Lee et al. 2018** [62]	rVE HD-QIV vs. SD-QIV -hospitalization	SLR with MA	MA of 7 RCTs and RW studies of HD-TIV vs. SD-TIV hospitalizations due to ILI in people 65+. No relevant risk of bias, moderate heterogeneity.	Another study was published later with similar inclusion criteria and different results.
**Puig-Barberá et al. 2016** [63]	rVE aTIV vs. SD-TIV	RW study	RW single-season study for rVE against hospitalization of virosomal-TIV vs. aTIV in Spanish population of 65+, mainly including lab-confirmed influenza	Virosomal-TIV was used as an approximation for SD-TIV because despite its supposedly higher immunogenicity, there is no evidence of it being more effective than SD-TIV in preventing influenza hospitalization
***Ruiz Aragón et al. 2022*** [34]				
***Not available***	VE for QIV	NA	NA	Although mentioned in the methods, no value nor citation are available for QIV absolute effectiveness
**Diaz Granados et al. 2014** [22]	rVE for HD-QIV vs. SD-QIV	RCT	RCT for HD-TIV vs. SD-TIV in people 65+ (*n*=31,989). Two seasons (2010-11 y 2011-12)	Robust RCT. Its results were assumed to be applicable to HD-QIV vs. SD-QIV based on an immune-bridging clinical trial as supported by EMA
***Ad-hocx MA*** [34]	rVE for aQIV vs. HD-QIV	MA	Non-statistically significant MA of 7 RW studies with very high heterogeneity	The MA was not based on an SLR
***Fochesato et al. 2022*** [30]				
**Belongia et al. 2021** [24]	VE for QIVe	SRL with MA	MA of 56 RW studies of VE with test-negative design and lab-confirmed influenza.	Test-negative design is only one of the available designs for evaluating VE; thus, not all available evidence is included here.
**Calabró et al. 2021** [25]	rVE for aQIV vs. QIVe	SRL with MA for HTA	RSL with MA of aTIV vs. TIV.	
***Ruiz Aragón et al. 2023*** [33]				
**Izurieta et al. 2021** [64]	rVE for QIVr vs. aQIV	RW study	RW study of the rVE of QIVr vs. aTIV in people 65+ (Medicare) in a single season (2019-20)	Season was truncated due to escalation of the COVID-19 pandemic

*aTIV* Adjuvated-TIV, *CDC* Centre for Disease Control, *CEA* Cost-effectiveness analysis, *EMA* European Medicines Agency, *GP* General practice, *ED* Emergency department, *HD-QIV* High-dose-QIV, *HTA* Health-technology assessment, *ILI* Influenza-like illness, *MA* Meta-analysis, *RCT* Randomized controlled study, *rVE* Relative vaccine effectiveness, *RW* Real World, *QIV* Quadrivalent inactivated vaccine, *QIVc* cell-based-QIV, *QIVe* egg-based-QIV, *QIVr* recombinant-QIV, *SD-TIV* Standard-dose-TIV, *SLR* Systematic literature review, *TIV* Trivalent inactivated vaccine, *VE* Vaccine effectiveness

#### Model uncertainty

WHO guidelines [13, 28] state that decision trees are appropriate to model short-term outcomes over one influenza season when the intervention is aimed at a population in whom vaccination is unlikely to change the infection dynamics (e.g. older adults). For populations that play a key role in infection transmission (e.g., children), dynamic models should be used. According to these recommendations, all studies included in our analysis used an appropriate model type. Specifically, two studies that include children in their target population used dynamic transmission models [29, 30] to account for *herd immunity*, and one used a static transition model [31]. The remaining studies used decision trees [32–35], which are appropriate considering the short-term time horizon used for the calculations (one influenza season or 1 year) and the exclusion of the paediatric population from their models. Health outcomes in the decision tree model are defined with sufficient clarity to include all the consequences of influenza infection (symptomatic cases, general practice visits, emergency department visits, inpatient stays and deaths).

#### Methodological uncertainty

All the models studied adequately define population, intervention, comparator, outcomes, and time horizon. Although, these aspects were chosen in accordance with the international [17, 36–39] and national [40] guidelines on economic evaluations, some merit additional consideration. Population was correctly defined based on the Spanish guidelines for influenza vaccination, which recommend vaccinating high-risk individuals of all ages and the older adults [6, 7], while three articles only considered older adults [33–35] which was consistent with the investigational question.

In CEEs, it is essential to clearly define the perspective of the analysis according to guidelines [36, 38], and to report the results separately in order to quantify the costs assumed by each payer. While most studies adopted the societal perspective [38, 40], this approach may not be particularly relevant to the decision-maker when the intervention is aimed at the aging (retired) population. In this regard, five studies [29–32, 34] used both the Spanish NHS and societal perspectives, one [35] chose to only use the Spanish NHS perspective, and another does not explain the perspective adopted, although it is reasonable to assume that they adopted the societal perspective because they include loss of productivity; however, not even this specific topic is clarified, and the NHS and societal perspective results are not presented separately [36, 40].

All included CEAs estimated the health benefits in terms of quality-adjusted life years (QALYs) as it is the priority outcome for reimbursement negotiations in Spain [41]; however, a few have also estimated them in life-years gained (LYG), as well as including a budget-impact model.

Finally, although the WHO recommends [13, 28] the inclusion of adverse event after immunization (AEFI) rates in influenza vaccine modelling when possible, none of the analysed studies have done so.

#### Parameter uncertainty

The systematic reviews with meta-analyses published so far have been used as sources for absolute vaccine effectiveness (VE) data (*n* = 5, of which one was a Cochrane review [42]). Interestingly, to inform relative effectiveness of comparators, one study [34] used an ad-hoc meta-analysis that was not based on a systematic search, while another study [30] used the result of a meta-analysis that had been measured in the framework of an Italian health-technology assessment and sponsored by the manufacturer [25]. In addition, two randomized-controlled trials (RCTs) and three observational studies used only single-season data.

Another key feature to take into consideration when reviewing effectiveness sources is the definition of influenza. While the most reliable way to diagnose influenza infection is laboratory confirmation using a previously established clinical definition, most studies have used the wider, less precise definition of influenza-like illness (ILI), thereby increasing the uncertainty of effectiveness data. Furthermore, two studies do not inform the influenza definition [33, 34]; another one [29] used only laboratory-confirmed influenza data, and the other four studies [30–32, 35] describe sources that used either definition (see Table 2).

The WHO guidance [13, 28] recommends averaging the burden of illness over at least 5 influenza seasons in order to minimize inter-seasonal variability of disease burden, and all CEAs used local epidemiological data. Nonetheless, three CEAs [32–34] used only three seasons, one used 1 season for general practice (GP, ) emergency department (ED), and hospitalization, and 6 seasons for deaths [31], and one does not specify the period studied [30]. Conversely, Crépey et al. 2021 [29] used five seasons and Redondo et al. 2021 [35] used eight, in compliance with best practices.

In cost-utility analyses, utilities are the final outcome that measures the effectiveness of the interventions analysed. All analysed studies use a Spanish study [43] to derive disutility associated with influenza, except for one [30], which uses Italian data.

#### Management of uncertainty

Deterministic one-way and probabilistic sensitivity analyses (DSA and PSA) were performed in all CEAs. Four [29, 31, 33, 34] studies did not fully report DSA variation intervals and/or PSA probability distributions. One study [32] used an arbitrarily chosen percentage variation of the baseline case value instead of using the measures of dispersion (e.g., confidence intervals) originally estimated from the model.

## Discussion

To our knowledge, this is the first, comprehensive, systematic analysis of all available evidence on the CEAs of influenza vaccines in the population aged ≥ 60 years in Spain. We used a systematic approach based on various different validated assessment tools to capture the magnitude of the uncertainty of the results [13, 14, 17, 19–21, 28, 44].

Our assessment showed that the CEAs published so far generally follow applicable guidelines [13, 17, 28, 36–40, 45, 46]. Nonetheless, we found that some decisions concerning the model structure, methods, and parameters of the models did not follow best practices. The derived uncontrolled uncertainty can thus yield non-convergent results that may hinder the decision-making process by adding confounding bias.

Effectiveness is one of the variables with the highest impact on the incremental cost-effectiveness ratio (ICER). VE is hard to determine, mainly due to the seasonal variation of circulating strains and the degree to which the vaccine formulated for each season matches the circulating virus strains in a specific territory. For this reason, the WHO recommends [28, 42] deriving VE from systematic reviews with meta-analyses, or using a range of values from RCTs and real-world studies that have undergone sensitivity analyses representative of extreme circumstances [13].

Very few RCTs in influenza vaccines for the elderly have been published in the past thirty years, although numerous observational studies are published annually [47]. Due to the high heterogeneity of study design, it is essential to carefully select the source of efficacy data. The reliability of cost-effectiveness evaluation (CEE) results hinges on the meticulous choice of effectiveness data. According to ISPOR guidelines, modelers should “seek to identify and incorporate all relevant evidence, rather than cherry picking the best single source of evidence for that parameter; use best practice methods to avoid potential biases in parameter estimates that might arise (for example, when estimating treatment effectiveness from observational sources); and employ formal evidence syntheses techniques (meta-analysis and network meta-analysis) as appropriate”.

Nonetheless, we have found that some studies are not transparent in their selection of VE data, and this casts doubts on their results. For example, in Ruiz-Aragón et al. 2020 [32] the reported source [27] of rVE data used to compare QIVc vs. QIVe was a presentation for an oral communication in a course that was not accessible [27, 32]. The authors do not explain or justify their reason for choosing this source and, as noted by Alvarez et al. 2021 [48], at least four other studies had been published at the time of the evaluation [49–52]. The use of single-season observational studies as a source of rVE data may be unavoidable due to the scarcity of more robust evidence. However, their use should be justified and put in the context of other available evidence. For example, the rVE of aTIV vs. SD-TIV in Redondo et al. 2021 [35] is derived from a Spanish single-season study, and the use of this source is thoroughly justified and contextualised in the methods section of the paper.

Deciding whether effectiveness results are correctly used in CEAs may be challenging for the average reader. For example, in Ruiz-Aragon et al. 2022, an ad-hoc meta-analysis was used to estimate the rVE of aQIV vs. HD-QIV. However, article compilation and review methodology are poorly described: half of the included studies were derived from a previous meta-analysis [26], and the other half were identified using a “targeted” non-systematic literature review. The estimated rVE of aTIV and HD-TIV was 4.0 (−0.05; 8.4), with considerable (*I*^*2*^ = 92%) heterogeneity (*p* < 0.1) [12]. While the authors conclude that aTIV and HD-TIV, and therefore aQIV and HD-QIV, are equally effective, no firm conclusions can be drawn due to the high degree of heterogeneity. Thus, the source of this heterogeneity should be investigated to assess whether it makes sense to combine studies, and a series of strategies, such as subgroup meta-analysis, can be used to reduce heterogeneity [12, 53]. For example, studies including GP outcomes could have been separated from those not including GP. However, the authors decided to use a random-effect method to meta-analyse the entire data set incorporating the “noise” from heterogeneity, but this did not compensate for the fact that different outcomes were included in the same average [53], and the results of the meta-analysis do not support any firm conclusions. In this respect, according to the ECDC [54], there is limited evidence of the higher effectiveness of adjuvated vaccines compared to standard doses against laboratory-confirmed influenza with rVE vs. SD ranging between − 30% (95%CI: −146 to 31%) and 88% (95%CI: 51 to 100) based on seven non-randomized studies of intervention (low certainty of evidence) [54]. Domnich et al. 2017 [55], attempting to synthesize the available efficacy data for aTIV, acknowledged that it was impossible to determine the rVE of aTIV vs. other vaccines due to the different designs, settings, comparators, and outcomes used in the studies analysed. Finally, in their RCT assessing aQIV efficacy vs. a non-influenza comparator, Beran et al. 2021 [56] were unable to meet their pre-specified primary efficacy endpoint. RCTs support the greater efficacy of high-dose vs. standard-dose vaccines in the elderly, and in observational studies they are generally more effective than standard-dose vaccines. Thus, according to the ECDC [54], the rVE estimate against laboratory-confirmed influenza was 24.2% (95%CI: 9.7 to 36.5%) in one RCT (moderate certainty of evidence).

Similar reasoning applies to studies [30, 33] using data from Coleman et al. 2021 [26], in which at least three of the included studies have a high risk of bias, and the resulting meta-analysis is highly heterogenous. As pointed out by Yin et al. 2021 [57], the combination of these factors “detracts from the value of Coleman’s analysis to inform clinical practice and policy recommendations” [30]. In summary, working with meta-analyses results requires careful assessment of the meta-analyses themselves in order to prevent introducing uncertainty and structural bias in the model.

In summary, when considering effectiveness data in a CEA, decision-makers should retrieve original data sources and critically consider the general study design, how influenza cases were defined, how many seasons did it include, if it was a meta-analysis, how high was heterogeneity between studies? Were the summary measures correctly derived and interpreted? Was the selected source the best available in general?

Fochesato et al. [30] compare aQIV to QIVe, not including other relevant comparators, such as HD-QIV. According to the authors, aQIV has already been shown to be more cost-effective than HD-QIV in the older adults in Spain [34]. However, Redondo et al. 2021 [35] had shown that HD-QIV is cost-effective compared to aQIV from the NHS perspective. This discrepancy in the evidence is not discussed by Fochesato et al. 2022, and HD-QIV and other potential comparators were not included in their model. Thus, as new vaccine options become available, multiple comparators may be important for decision-making, especially when previous studies have reported contradictory results. Therefore, comparator selection should be clearly justified [13, 28, 38].

The WHO guidelines recommend estimating burden of illness by averaging at least 5 seasons. Although the Spanish Influenza Surveillance System provides data for as many seasons as desired, only two CEAs [29, 35] have averaged 5 seasons. It is reasonable to assume that the more seasons used, the more robust the estimated burden-of-illness.

In dynamic models, the modelling of contact maps and rates is crucial. In Fochesato et al. 2022, Italian data were used due to the lack of Spanish data. The Italian data were considered transferable to Spanish models when at least one of the viral strains was circulating in both Italy and Spain in the same season. However, the coinciding strain is often H1N1, against which all vaccines are highly effective in the elderly [58], while in four seasons either H3N2 or B (or both) were present in one of the two countries and absent in the other. Thus, Italian data cannot be transferred to the Spanish model. Furthermore, since model calibration was not reported, it is impossible to determine the extent to which Italian data reproduce Spanish epidemiology.

According to specific guidelines, adverse events (AE) should be included in economic models of influenza vaccines [13, 28], but none of the analysed CEAs has done so, and none have explained this omission, although it is probably due to the generally perceived good safety profile of influenza vaccines and the difficulty in estimating the cost of such adverse events in Spain. From a 2021 systematic review [59] that gathered and analysed the inclusion of safety-related issues in economic evaluations for seasonal influenza vaccine, it emerges that, over the included 52 economic evaluations, the consequences of AEFIs were included as: direct and indirect costs in 90% and 27% of cases, an disutilities in 37% of them. Severe AEFI (GBS, anaphylaxis, and MSW) were included in 33% of studies, minor/mild AEFI (local and systemic) in 46%, and 40% of studies did not specify the considered type of AEFI.

To sum up, apart from the typical aspects of a CEA (perspective, time-horizon, discounting, etc.) decision-makers should pay attention to such parameters as the number of influenza seasons used to estimate disease burden, the transferability of parameters derived from other settings, the accounting for adverse events both in terms of cost and disutility,

Finally, sensitivity and scenario analyses are key features of parameter uncertainty. This particularly affects the reader’s evaluation of the impact of parameters showing significant uncertainty.

Thus, a lack of proper uncertainty management as well as insufficient transparency in presenting and justifying design and parameter choice should alert the critical reader and warrant a deeper investigation of the quality of the CEA.

This study has limitations inherent to its design. First, a certain degree of subjectiveness is unavoidable. To overcome this, the study was performed by a multidisciplinary team made up of clinicians from all therapeutic areas involved in influenza prevention, as well as experts in the field of pharmacoeconomics. Second, there are no “rules” that can be applied to show whether a study has been well conducted and to rate the seriousness of deviations from published recommendations. Although we used TRUST to analyse the relevant CEAs, this tool is not straightforward. To overcome this, we reviewed the applicable recommendations of official organizations such as ISPOR, WHO and national institutions, and constructed a common theoretical framework that all researchers involved could use to perform an objective, comprehensive, critical analysis. The primary aim of this analysis was to provide a descriptive overview of the current landscape of CEAs for influenza vaccines in the elderly population in Spain. Based on the insights gained from this review, we are in the process of developing a practical guide for decision-makers. This guide, which will be available soon, is designed to help readers independently identify and assess uncertainties in influenza CEAs, enabling more informed decision-making.

## Conclusion

A number of CEAs assessing the cost-effectiveness of influenza vaccines have been published in recent years. To properly leverage the result of these studies, we must never forget that we deal with estimates deriving from modelling techniques. Thus, due to the challenges inherent to modelling, uncertainty management is crucial, particularly when analysing variables such as effectiveness. Justifying the selection of primary clinical data sources and openly discussing their limitations is essential for reader interpretation. However, healthcare professionals and decision-makers should be aware that “all that glitters is not gold” in economic evaluations, and transparent, comprehensive information on methods and results, together with careful critical reading, are paramount in evidence-based medicine.

## Supplementary Information

Supplementary Material 1.

Supplementary Material 2.

Supplementary Material 3.

Supplementary Material 4.

Supplementary Material 5.

Supplementary Material 6.

Supplementary Material 7.

Supplementary Material 8.

Supplementary Material 9.

Supplementary Material 10.

Supplementary Material 11.

Supplementary Material 12.

## Data Availability

All study materials are available in the additional files.

## Abbreviations

AMSTAR-2: A Measurement Tool to Assess Systematic Reviews-2
aQIV: adjuvated-Quadrivalent Influenza Vaccine
aTIV: adjuvated Trivalent Influenza Vaccine
CEA: Cost-Effectiveness Analysis
CEE: Complete Economic Evaluation
DSA: Deterministic Sensitivity Analysis
ED: Emergency Department
GP: General Practice
HD-QIV: High-Dose Quadrivalent Influenza Vaccine
NHS: National Health Service
ICER: Incremental Cost-Effectiveness Ratio
NOS: Newcastle-Ottawa Scale
PRISMA: Preferred Reporting Items for Systematic Review and Meta-Analysis
PSA: Probabilistic Sensitivity Analysis
QIV: Quadrivalent Influenza Vaccine
QIVe: egg-based Quadrivalent Influenza Vaccine
QIVc: cell-based Quadrivalent Influenza Vaccine
QIVr: Recombinant Quadrivalent Influenza Vaccine
RCT: Randomized-Controlled Trial
RoB 2: Risk of bias 2
rVE: relative Vaccine Effectiveness
SD-QIV: Standard-Dose Quadrivalent Influenza Vaccine
TIV: Trivalent Influenza Vaccine
TRUST: TRansparent Uncertainty ASsessmenT
WHO: World Health Organization
WTP: Willingness-To-Pay
