# Supplementary material for: Critical assessment of uncertainty in economic evaluations on influenza vaccines for the elderly population in Spain
Source: BMC Infect Dis. 2025 Feb 1;25:152. doi: 10.1186/s12879-025-10442-3 (PMC11786407; doi:10.1186/s12879-025-10442-3)
Supplement: Supplementary file 1 — Supplementary Material 1. [file 12879_2025_10442_MOESM1_ESM.docx]

# A1. Systematic review details

## A.1.1 Investigation question

“Which is the cost-effectiveness of influenza vaccines for people 60-or-more-year-old in Spain?”

## A.1.2 PICO question

| Patient | Spanish adult population starting from 60 year old |
| --- | --- |
| Intervention | Influenza vaccines |
| Comparator | Influenza vaccines |
| Outcomes | Incremental Cost-Effectiveness or Cost-Utility Ratio (ICER or ICUR) |

## A1.3 Search terms and synomims

| Nº. | Término | Sinónimos |
| --- | --- | --- |
| #1 | Spanish population | Spanish OR Spain |
| #2 | Influenza vaccine | ("Influenza, Human"[Mesh] OR (influenza) OR flu) AND (vaccin* OR immunization) |
| #3 | Economic evaluations | ("Cost-Benefit Analysis"[Mesh] OR (cost-effectiveness) OR (cost-utility) OR (economic evaluation)) |

## A1.4 Search strategy

1. #1 AND #2 AND #3

Table A1. Complete search string for each database

| N.º | PubMed | Scopus | WoS | BVS |
| --- | --- | --- | --- | --- |
|  | ("Cost-Benefit Analysis"[Mesh] OR (cost-effectiveness) OR (cost-utility) OR (economic evaluation)) AND ("Influenza, Human"[Mesh] OR (influenza) OR flu) AND (vaccin* OR immunization) AND (spain OR spanish) | ((cost-effectiveness) OR (cost-utility) OR (economic evaluation)) AND (influenza OR flu) AND (vaccin* OR immunization) AND (spain OR spanish) | ((cost-effectiveness) OR (cost-utility) OR (economic evaluation)) AND (influenza OR flu) AND (vaccin* OR immunization) AND (spain OR spanish) | ((cost?-efectividad) OR (cost?-utilidad)) AND gripe AND (vacuna* OR inmunización) AND (españa OR español) |

## A1.5 Selection criteria

## Inclusion criteria:

## Original publications in National or International peer-reviewed journals;

## CEE of influenza vaccines including population ≥60 years old, in Spain;

- - Outcomes: Life years gained (LYG) or Quality-Adjusted Life Years (QALYs) or monetary benefits in case of Cost-benefit Analysis;
  - Language: Spanish or English.

## Exclusion criteria:

## Incomplete economic-evaluations (those evaluations that do not include the analysis of both costs and benefits, e.g.., budget-impact models).

## Letters, editorials, expert opinion, abstracts, doctoral theses.

## Original studies on the cost-effectiveness or cost-utility of vaccine programs (vaccine strategies, not single vaccines).

## Systematic reviews, if any, would be excluded after reviewing the included primary references, when they had not appeared in the search.
