# Supplementary material for: Critical assessment of uncertainty in economic evaluations on influenza vaccines for the elderly population in Spain
Source: BMC Infect Dis. 2025 Feb 1;25:152. doi: 10.1186/s12879-025-10442-3 (PMC11786407; doi:10.1186/s12879-025-10442-3)
Supplement: Supplementary file 2 — Supplementary Material 2. [file 12879_2025_10442_MOESM2_ESM.docx]

# A2. Selection process

Figure A1. PRISMA workflow of reference selection

Records identified from subsequent non-systematic database search

(n = 1)

Records removed *before screening*:

Duplicate records removed (n = 69)

Records identified from*:

Databases (n = 163)

**Identification**

Records excluded

(n = 85)

Records screened

(n = 94)

**Screening**

Reports sought for retrieval

(n = 9)

Reports not retrieved

(n = 0)

Reports assessed for eligibility

(n = 9)

Reports excluded:

Bugdet impact analysis: n = 3

Studies included in review

(n = 6)

Studies included

(n= 1)

**Included**

Table A2. Excluded references with reason

| Referencia | Título | Motivación |
| --- | --- | --- |
| Ruiz-Aragón et al. 2015 | Estimate of the impact of influenza vaccination with adjuvant MR59 in population over 64 years for the National Health System: Effects and costs | Budget Impact Model |
| Uhart et al. 2016 | Public health and economic impact of seasonal influenza vaccination with quadrivalent influenza vaccines compared to trivalent influenza vaccines in Europe | Budget Impact Model |
| Pérez-Rubio et al., 2018 | Economic and Health impact of influenza vaccination with adjuvant MF59 in population over 64 years in Spain | Budget Impact Model |
