# Supplementary material for: Critical assessment of uncertainty in economic evaluations on influenza vaccines for the elderly population in Spain
Source: BMC Infect Dis. 2025 Feb 1;25:152. doi: 10.1186/s12879-025-10442-3 (PMC11786407; doi:10.1186/s12879-025-10442-3)
Supplement: Supplementary file 3 — Supplementary Material 3. [file 12879_2025_10442_MOESM3_ESM.docx]

# A3. Quality of evidence

## A3.1 Description of the analysis tool employed

- **The TRUST Tool (Tool for Evaluating Research Use in Systematic Reviews)**^1^: a validated structured framework that assesses the use of evidence economic evaluations, with a focus on addressing various types of uncertainty. It evaluates **structural**, **methodologic**, and **parametric uncertainty**—ensuring transparency and rigor by assessing biases, model assumptions, data variability, and other factors critical to healthcare decision-making.
- **The WHO “Guide for standardization of economic evaluations of immunization programmes Geneva: World Health Organization^2^” and “WHO guidance on the economic evaluation of influenza vaccination”^3^**: provides a standardized approach to the economic evaluation of influenza vaccines and influenza vaccination programs. It outlines methods for assessing cost-effectiveness, budget impact, and broader economic benefits to support decision-makers in prioritizing vaccination strategies in global health policies.
- **ISPOR (International Society for Pharmacoeconomics and Outcome Research) Task force documents^4-9^:** consensus documents developed by expert groups that provide guidance on best practices in health economics and outcomes research, covering a wide range of topics, including economic evaluations, real-world evidence, patient-reported outcomes, and modeling techniques. Their recommendations aim to improve methodological rigor, transparency, and consistency in research, supporting evidence-based healthcare decision-making worldwide.
- **The Spanish Health Ministry “Guidance for the economic evaluation of drugs”^10^**: the national guidance for the development of economic evaluations to be used for the reimboursment negotiations with the Spanish Ministry of Health.
- **RoB 2 (Risk of Bias 2 Tool)^11^:** a revised tool for assessing the risk of bias in randomized controlled trials. It focuses on five domains of bias, including randomization process, deviations from intended interventions, missing outcome data, measurement of the outcome, and selection of the reported result. It provides a structured approach to evaluate methodologic uncertainty and enhance the reliability of evidence.
- **AMSTAR 2 (A Measurement Tool to Assess Systematic Reviews 2)^12^:** an updated tool for appraising the quality of systematic reviews, specifically those of randomized and non-randomized studies. It evaluates key domains such as protocol registration, search strategy, and data synthesis, addressing methodologic and structural uncertainty to ensure systematic reviews are both comprehensive and robust.
- **NOS (Newcastle-Ottawa Scale)^13^:** a widely used tool for assessing the quality of non-randomized studies, particularly cohort and case-control studies. It focuses on three key domains: selection, comparability, and outcome/exposure, addressing parametric and methodologic uncertainty by evaluating study design, participant selection, and the robustness of the results.

## A3.2 Quality of evidence of the primary sources of efficacy/effectiveness used in the CEAs

Table A3 shows a summary of the quality check of the primary sources of efficacy/effectiveness used in each CEA. It reports the validated checklist that was applied according to the study design (RoB2 for clinical trials, NOS for real-world studies, and AMSTAR-2 for systematic reviews with meta-analysis). The risk of bias resulting from the analysis is reported as well as its interpretation according to each validated tool (NOS does not provide any further interpretation, thus NA was noted). All sources are reported for completeness; however, only those directly regarding elderly people were evaluated. This analysis provided an external judgement of the quality of the source according to another validated tool.

Table A3. Summary of the quality of evidence of the primary efficacy/effectiveness sources used as input in the included economic evaluations

| Reference | Checklist | Qyality of the source | Risk of bias judgement | Comments |
| --- | --- | --- | --- | --- |
| *Garcia et al. 2016* | | | | |
| Tricco et al, 2013^14^ | AMSTAR-2 | Critically low quality review | High | More than one critical flaw with or without non-critical weaknesses: the review has more than one critical flaw and should not be relied on to provide an accurate and comprehensive summary of the available studies |
| *Crépey et al. 2020* | | | | |
| DíazGranados 2012^15^ | - | Not evaluated | NA | The age range of this study is out of scope |
| Center for Disease Control | - | Not evaluated | NA | Incomplete citation, source not available for evaluation |
| *Ruiz-Aragón et al. 2020* | | | | |
| Belongia et al. 2016^16^ | AMSTAR-2 | Critically low quality review | High | More than one critical flaw with or without non-critical weaknesses: the review has more than one critical flaw and should not be relied on to provide an accurate and comprehensive summary of the available studies |
| Boikos et al. 2018 | NOS | Not evaluated | NA | This source was not publicly available (congress communication, only abstract accessible) |
| *Redondo et al. 2021* | | | | |
| Govaert 1994 | ROB-2 | Some concerns | Moderate | The study is judged to raise some concerns in at least one domain [Domain 5] for this result, but not to be at high risk of bias for any domain. |
| DíazGranados 2014^17^ | ROB-2 | High | Low | Low risk of bias |
| DíazGranados 2012^15^ | - | Not evaluated | NA | The age range of this study is out of scope |
| Lee 2018^18^ | AMSTAR-2 | Low quality review | Moderate | One critical flaw with or without non-critical weaknesses: the review has a critical  flaw and may not provide an accurate and comprehensive summary of the available  studies that address the question of interest |
| Puig-Barberà 2013^19^ | NOS | Good (8 stars) | Low | NA |
| *Ruiz-Aragón et al. 2022* | | | | |
| Ruiz-Aragón 2022^20^ | Not aplicable | Not evaluated | NA | Non-systematic review of which 4 studies were selected |
| Coleman 2021^21^ | AMSTAR-2 | Not evaluated | NA | Only 4 of the studies retrieved in this systematic review were selected |
| *Fochesato et al. 2022* | | | | |
| Boccalini 2021^22^ | AMSTAR-2 | Not evaluated | NA | The age range of this study is out of scope |
| Calabrò 2021^23^ | AMSTAR-2 | Critically low quality review | High | More than one critical flaw with or without non-critical weaknesses: the review has more than one critical flaw and should not be relied on to provide an accurate and comprehensive summary of the available studies |
| Coleman 2021^21^ | AMSTAR-2 | Critically low quality review | High | More than one critical flaw with or without non-critical weaknesses: the review has more than one critical flaw and should not be relied on to provide an accurate and comprehensive summary of the available studies |
| *Ruiz Aragon et al. 2023* | | | | |
| Izurieta et al. 2021^24^ | NOS | Good (8 stars) | Low | NA |

*RoB-2: Risk of Bias-2; NOS: Newcastel-Ottawa Score; AMSTAR-2: Assessing the Methodologic Quality of Systematic Reviews-2; NA: not applicable*

**REFERENCES**

1. Grimm SE, Pouwels X, Ramaekers BLT, et al. Development and Validation of the TRansparent Uncertainty ASsessmenT (TRUST) Tool for Assessing Uncertainties in Health Economic Decision Models. *Pharmacoeconomics*. Feb 2020;38(2):205-216. doi:10.1007/s40273-019-00855-9

2. Organization WH. *WHO guide for standardization of economic evaluations of immunization programmes*. 2019.

3. Newall AT, Chaiyakunapruk N, Lambach P, Hutubessy RCW. WHO guidance on the economic evaluation of influenza vaccination. *Influenza and Other Respiratory Viruses*. 2018;12(2):211-219. doi:<https://doi.org/10.1111/irv.12510>

4. Briggs AH, Weinstein MC, Fenwick EA, Karnon J, Sculpher MJ, Paltiel AD. Model parameter estimation and uncertainty analysis: a report of the ISPOR-SMDM Modeling Good Research Practices Task Force Working Group–6. *Medical decision making*. 2012;32(5):722-732.

5. Caro JJ, Briggs AH, Siebert U, Kuntz KM. Modeling good research practices--overview: a report of the ISPOR-SMDM Modeling Good Research Practices Task Force--1. *Value Health*. Sep-Oct 2012;15(6):796-803. doi:10.1016/j.jval.2012.06.012

6. Eddy DM, Hollingworth W, Caro JJ, Tsevat J, McDonald KM, Wong JB. Model transparency and validation: a report of the ISPOR-SMDM Modeling Good Research Practices Task Force-7. *Med Decis Making*. Sep-Oct 2012;32(5):733-43. doi:10.1177/0272989x12454579

7. Pitman R, Fisman D, Zaric GS, et al. Dynamic transmission modeling: a report of the ISPOR-SMDM Modeling Good Research Practices Task Force Working Group-5. *Med Decis Making*. Sep-Oct 2012;32(5):712-21. doi:10.1177/0272989x12454578

8. Roberts M, Russell LB, Paltiel AD, Chambers M, McEwan P, Krahn M. Conceptualizing a model: a report of the ISPOR-SMDM Modeling Good Research Practices Task Force-2. *Med Decis Making*. Sep-Oct 2012;32(5):678-89. doi:10.1177/0272989x12454941

9. Siebert U, Alagoz O, Bayoumi AM, et al. State-transition modeling: a report of the ISPOR-SMDM modeling good research practices task force-3. *Value in Health*. 2012;15(6):812-820.

10. Comité Asesor para la Financiación de la Prestación Farmacéutica del Sistema Nacional de Salud. *Guía de evaluación económica de medicamentos.* 2023.

11. Sterne JA, Savović J, Page MJ, et al. RoB 2: a revised tool for assessing risk of bias in randomised trials. *bmj*. 2019;366

12. Shea BJ, Reeves BC, Wells G, et al. AMSTAR 2: a critical appraisal tool for systematic reviews that include randomised or non-randomised studies of healthcare interventions, or both. *bmj*. 2017;358

13. Wells G, Shea B, O’Connell D, et al. Newcastle-Ottawa quality assessment scale cohort studies. *University of Ottawa*. 2014;

14. Tricco AC, Chit A, Soobiah C, et al. Comparing influenza vaccine efficacy against mismatched and matched strains: a systematic review and meta-analysis. *BMC Med*. Jun 25 2013;11:153. doi:10.1186/1741-7015-11-153

15. DiazGranados CA, Denis M, Plotkin S. Seasonal influenza vaccine efficacy and its determinants in children and non-elderly adults: A systematic review with meta-analyses of controlled trials. *Vaccine*. 2012/12/17/ 2012;31(1):49-57. doi:<https://doi.org/10.1016/j.vaccine.2012.10.084>

16. Belongia EA, Simpson MD, King JP, et al. Variable influenza vaccine effectiveness by subtype: a systematic review and meta-analysis of test-negative design studies. *Lancet Infect Dis*. Aug 2016;16(8):942-51. doi:10.1016/s1473-3099(16)00129-8

17. DiazGranados CA, Dunning AJ, Kimmel M, et al. Efficacy of High-Dose versus Standard-Dose Influenza Vaccine in Older Adults. *New England Journal of Medicine*. 2014;371(7):635-645. doi:10.1056/NEJMoa1315727

18. Lee JKH, Lam GKL, Shin T, et al. Efficacy and effectiveness of high-dose versus standard-dose influenza vaccination for older adults: a systematic review and meta-analysis. *Expert Review of Vaccines*. 2018/05/04 2018;17(5):435-443. doi:10.1080/14760584.2018.1471989

19. Puig-Barberà J, Natividad-Sancho A, Calabuig-Pérez J, et al. MF59-adjuvanted and virosomal influenza vaccines for preventing influenza hospitalization in older people: Comparative effectiveness using the Valencia health care information system. *Vaccine*. 2013/08/20/ 2013;31(37):3995-4002. doi:<https://doi.org/10.1016/j.vaccine.2013.05.070>

20. Ruiz-Aragón J, Márquez-Peláez S, Gani R, Alvarez P, Guerrero-Luduena R. Cost-Effectiveness and Burden of Disease for Adjuvanted Quadrivalent Influenza Vaccines Compared to High-Dose Quadrivalent Influenza Vaccines in Elderly Patients in Spain. *Vaccines (Basel)*. Jan 23 2022;10(2)doi:10.3390/vaccines10020176

21. Coleman BL, Sanderson R, Haag MDM, McGovern I. Effectiveness of the MF59-adjuvanted trivalent or quadrivalent seasonal influenza vaccine among adults 65 years of age or older, a systematic review and meta-analysis. *Influenza Other Respir Viruses*. Nov 2021;15(6):813-823. doi:10.1111/irv.12871

22. Boccalini S, Pariani E, Calabrò GE, et al. [Health Technology Assessment (HTA) of the introduction of influenza vaccination for Italian children with Fluenz Tetra(®)]. *J Prev Med Hyg*. Jun 2021;62(2 Suppl 1):E1-e118. Health Technology Assessment (HTA) dell’introduzione della vaccinazione antinfluenzale per la popolazione giovanile italiana con il vaccino Fluenz Tetra(®). doi:10.15167/2421-4248/jpmh2021.62.2s1

23. Calabrò GE, Boccalini S, Bonanni P, Bechini A, Panatto D, Lai PL. Valutazione di Health Technology Assessment (HTA) del vaccino antinfluenzale quadrivalente adiuvato: Fluad Tetra. *Ital J Public Health*. 2021;10:97-122.

24. Izurieta HS, Lu M, Kelman J, et al. Comparative Effectiveness of Influenza Vaccines Among US Medicare Beneficiaries Ages 65 Years and Older During the 2019-2020 Season. *Clin Infect Dis*. Dec 6 2021;73(11):e4251-e4259. doi:10.1093/cid/ciaa1727
