# Supplementary material for: Critical assessment of uncertainty in economic evaluations on influenza vaccines for the elderly population in Spain
Source: BMC Infect Dis. 2025 Feb 1;25:152. doi: 10.1186/s12879-025-10442-3 (PMC11786407; doi:10.1186/s12879-025-10442-3)
Supplement: Supplementary file 4 — Supplementary Material 4. [file 12879_2025_10442_MOESM4_ESM.docx]

# A4. Uncertainty analysis

## A4.1 Detailed uncertainty analysis summary by CEA

1. ***García et al. 2016***

**a.1) *Summary of the study***

The present study estimates the cost-effectiveness of replacing TIVs with QIV for risk groups and elderly population in Spain. A static, lifetime, multi-cohort Markov model with a one-year cycle was adapted to assess the costs and health outcomes associated with a vaccine switch from TIV to QIV. The model followed a cohort vaccinated each year according to health authority recommendations, for the duration of their lives. National epidemiological data allowed the determination of whether the B strain included in TIVs matched the circulating one. Societal perspective was considered, costs and outcomes were discounted at 3% and one-way and probabilistic sensitivity analyses were performed. Compared to TIVs, QIV reduced more influenza cases and influenza-related complications and deaths during periods of B-mismatch. The incremental cost-effectiveness ratio (ICER) was 8,748€/quality-adjusted life year (QALY). One-way sensitivity analysis showed mismatch with the B lineage included in the TIV was the main driver for ICER. Probabilistic sensitivity analysis shows ICER below 30,000€/QALY in 96% of simulations. Replacing TIVs with QIV in Spain could improve influenza prevention by avoiding B virus mismatch and provide a cost-effective healthcare intervention.

***a.2) Model uncertainty***

- No relevant uncertainties were detected. The model type was adequately chosen.

***a.3) Parametric uncertainty***

- As for vaccine effectiveness (VE), VE of the three common influenza strains in the elderly population was based on the Cochrane review and meta-analysis by Jefferson et al. 2010. However, the authors already concluded that the meta-analysis results may have limited reliability due to the moderate risk of bias of some of the included studies.
- The effectiveness of the non-matching B strain for TIV the one of the TIV in case of mismatching season, and for QIV the one of TIV in case of complete matching season derived from a meta-analysis of RCTs by Tricco et al. 2013. This meta-analysis raises some questions because various studies included had RR overlapping with the RR=1. Also, heterogeneity in adults was generally reported as <50%, which may be considered moderate.

***a.4) Methodological uncertainty***

- WHO guidelines recommend basing disease burden on at least 5 influenza seasons to minimize interseason variability. In this study, general practice (GP) and emergency department (ED) burden of illness were estimated on just one season; however, hospitalization and death, which are economically more relevant events, were estimated based on 6 seasons.
- This study included at risk population of all ages. When productivity loss was considered, only direct productivity loss of workers is estimated; however, informal caregivers need to stay home to care for ill children, for which productivity loss should also be accounted for.
- Although WHO recommends^4,16^ including adverse events in the influenza vaccine modelling, none of the analyzed studies has done so. This is possibly due to the difficulty of estimating adverse events cost and to the general safety of influenza vaccines.

***a.5) Management of uncertainty***

- Deterministic and Probabilistic sensitivity analyses (DSA and PSA) were performed; however, neither the lower and upper bounds for DSA, nor the probability distributions used in PSA were disclosed, while it is expected that these are fully presented.

1. ***Crépey et al. 2020***

***b.1)* *Summary of the study***

A dynamic transmission model was developed to estimate the number of influenza B cases prevented under TIV and QIV strategies (<65 years (high risk) and ≥65 years). This model considers cross-protective immunity induced by different lineages of influenza B. The output of the transmission model was used as input for a decision-tree model that estimated the economic impact of switching TIV to QIV. The models were populated with Spanish data whenever possible. Replacing TIV with QIV in all eligible patients with current vaccine coverage in Spain may have prevented 138,707 influenza B cases per season and, therefore avoided 10,748 outpatient visits, 3,179 hospitalizations and 192 deaths. The replacement could save €532,768 in outpatient visit costs, €13 million in hospitalization costs, and €3 million in costs of influenza-related deaths per year. An additional €5 million costs associated with productivity loss could be saved per year, from the societal perspective. The budget impact from societal perspective would be €6.5 million, and the incremental cost-effectiveness ratio (ICER) €1,527 per quality-adjusted life year (QALY). Sensitivity analyses showed robust results. In additional scenarios, QIV also showed an impact at public health level reducing influenza B related cases, outpatient visits, hospitalizations and deaths.

***b.2) Model uncertainty***

- No relevant uncertainties were detected. The model type was adequately chosen.

***b.3) Parametric uncertainty***

- Effectiveness data are described as derived from a combination between the meta-analysis form DiazGranados et al. 2012, and data from the Centers for Disease Control (CDC). The methods used to derive these data are not explained; however, all data are transparently reported in the input table of the article, and authors affirm that they are similar to those reported in other studies.
- As for the cost of treatment, healthcare resource costs (GP and ED visits) were derived from eSalud, which is a database of Spanish Health costs. It includes multiple entries for each type of cost from different sources (official bulletins of the various autonomous communities, data from published studies etc.). Thus, for cost information to be complete, it would have been desirable that specific original sources of data were detailed, as well as the mathematical treatment used to obtain a single value.

***b.4) Methodological uncertainty***

- This study included at risk population of all ages. When productivity loss was considered, only direct productivity loss of workers is estimated; however, informal caregivers need to stay home to care for ill children, for which productivity loss should also be accounted for.
- Although WHO recommends^4,16^ including adverse events in the influenza vaccine modelling, none of the analyzed studies has done so. This is possibly due to the difficulty of estimating adverse events cost and to the general safety of influenza vaccines.

***b.5) Management of uncertainty***

- DSA variation intervals and PSA probability distributions were not informed. PSA is not represented as an acceptability curve, however the estimated ICER is clearly below the commonly referenced Spanish cost-effectiveness threshold for the base-case.

1. ***Ruiz Aragón et al. 2020***

***c.1) Summary of the study***

Author used a health economic model adapted to Spain to assess the costs-effectiveness of using quadrivalent cell-based inactivated vaccine (QIVc) instead of the egg-based vaccine (QIVe) in people aged 9–64 at high-risk of complications. Observed vaccine coverage of 32% in the 9–17 age group, 17% in those aged 18–59, and 22% for ages 60–64 was used in the analysis. In total, 2.5 million people were vaccinated in the simulations. The analysis includes the public payer and societal perspectives. All costs and outcomes are calculated for an entire influenza season, except for productivity and QALY loss due to premature death, which are calculated over the lifetime of the cohort, and discounted at 3% per year. Using QIVc instead of QIVe was associated with 16,221 fewer symptomatic cases, 4,522 fewer primary care visits, 1,015 fewer emergency room visits and 88 fewer hospitalizations. From a societal perspective, QIVc was more effective and less expensive compared to QIVe, leading to a cost-saving of €3.4 million. From a public payer perspective, the incremental cost-effectiveness ratio for QIVc vs QIVe was €12,852 per QALY gained. Authors conclude that QIVc offers a cost-effective alternative to QIVe and should be considered for people aged 9–64 at high-risk of influenza complications.

***c.2) Model uncertainty***

- No relevant uncertainties were detected. The model type was adequately chosen.

***c.3) Parametric uncertainty***

- How vaccine effectiveness (VE) data for the two alternatives were derived from the cited sources is poorly described in the article. VE data for QIVc refers to FLUCELVAX® QUADRIVALENT influenza vaccine.
- The QIVe VE was derived from Belongia et al. 2021, which provides VE for different strains by age group relative to “no vaccination”. VE for QIVc against the A/H3N2 strain, was also derived from Belongia et al. 2021. This is a systematic review and meta-analysis of 56 observational studies, including general population attending primary care for influenza. Test-negative design and influenza confirmation by PCR were the most relevant selection criteria for study inclusion. This may be a limitation, as other appropriate study designs, such as case-control or cohort studies, were not included.
- The same VE for strains A/H1N1 and B were used for both QIVc and QIVe. For A/H3N2, relative VE (rVE) between QIVc and QIVe was used to estimate VE for QIVc.
- rVE was derived from an observational study carried out in an adult population that estimated it for ILI cases. The cited source was a communication in a course about the rVE of QIVc vs. QIVe in a 17–64-year-old population in the season 2017-2018, and it is not publicly available. Also, the rVE originally estimated for a 18-64 population, was in Ruiz-Aragón also applied to the 9-17 age group. Nevertheless, in a subsequently published article regarding the following influenza season (2018-19), rVE was 3.9% (95% CI, .9–7.0) for the 4–17-year group, and 6.5% (95% CI, 5.2–7.9) for the 18–64 year group. In view of the assumptions that had to be made to apply Boikos et al. 2018 data to the model, the reason for choosing this source is not clear. In fact, as noted by Alvarez et al. 2021, at least four other studies had been published at the time of the evaluation.
- When considering utility values, when Spanish disutility data were available they were used; nevertheless, the baseline utility upon which they are applied are not mentioned. Finally, Vaccine coverage has been taken from data-on-file provided by Seqirus, while vaccination coverage is publicly available from official Spanish sources.

***c.4) Methodological uncertainty***

- In this study, the burden of illness was based on three influenza seasons, while WHO guidelines recommend that at least 5 seasons should be taken into account to control for interseasonal variations. Since the data source was the Spanish Influenza Surveillance System that has been issuing annual reports for the last 20 years, the reason why more seasons were not included is unclear.
- The description of how unit resource costs (GP ad ED visits) were derived is not clear in the report. Primary sources are both national and local official bulletins. In Spain, these tariffs are highly variable across autonomous communities; thus, the reason for choosing a particular subset of sources and how the corresponding values have been mathematically treated to obtain a single value, should be explained.
- Although WHO recommends^4,16^ including adverse events in the influenza vaccine modelling, none of the analyzed studies has done so. This is possibly due to the difficulty of estimating adverse events cost and to the general safety of influenza vaccines.

***c.5) Management of uncertainty***

- Both PSA and DSA have been carried out; however, probability distributions used in the PSA have not been reported. In DSA, the base case value has been arbitrarily varied by ±10%, in contrast with guidelines.

1. ***Redondo et al. 2021***

***d.1) Summary of the study***

This was a decision-tree model estimating the cost-utility of HD-QIV and aTIV for influenza vaccination in the Spanish population ≥ 65 years old from the NHS perspective. Symptomatic influenza cases, visits to the general practitioner (GP), visits to the emergency department (ED), hospitalizations, and mortality related to influenza were considered. Results showed that switching from aTIV strategy to HD-QIV would prevent 36,476 cases of influenza, 5,143 visits to GP, 1,054 visits to the ED, 9,193 episodes of hospitalizations due to influenza or pneumonia, and 357 deaths due to influenza. This would result in 3,514 life-years and 3,167 quality–adjusted life–years (QALYs) gained. Healthcare costs increased by €78,874,301, leading to an incremental cost-effectiveness ratio of €24,353/QALY. The sensitivity analysis indicates that the results are rather robust. Therefore, authors conclude that HD-QIV would be cost-effective compared to aTIV in the population under study.

***d.2) Model uncertainty***

- No relevant uncertainties were detected. The model type was adequately chosen.

***d.3) Parametric uncertainty***

- Given there was no head-to-head evidence comparing rVE between HD-QIV and aTIV, rVE was derived from indirect comparison through a common comparator, SD-TIV. Absolute effectiveness for SD-TIV was assigned on the grounds of an RCT, Govaert et al. 1994.^40^ SD-QIV was considered to be as effective as SD-TIV for the three common strains, and its effectiveness in the second B strain has been assumed to be similar to that established in other age-groups.^41^
- The rVE of HD-QIV vs. SD-QIV was assumed to be the same as HD-TIV vs. SD-TIV assessed by the FIM12 RCT ^42,43^, which is justified by an immune-bridging clinical trial ^44^, an approach that has been approved by the European Medicines Agency (EMA).^45^ Thus, it has been derived from the RCT by DiazGranados et al. 2014.^42^
- Conversely, there was no evidence showing superiority of aTIV versus SD-TIV in an RCT in individuals aged ≥ 65 years. Considering no robust evidence was available for rVE of aTIV vs. SD-TIV, the base case scenario should have considered a 0% relative effectiveness, i.e., non-inferiority hypothesis between comparators. However, authors considered that this was unlikely to be real, thus they derived the rVE of aTIV vs. SD-TIV against hospitalization from an observational study^46^ which was the only one available in Spain in a post-pandemic season being carried out in elderly population. This study based on a regional vaccination database with low risk of bias, including mainly lab-confirmed influenza cases (84%), had been considered as a good level of evidence by NACI and low risk of bias by ECDC. However, its main limitation is that it only collected data from a single influenza season, which moreover was characterized by high vaccine matching due to the main circulation of H1N1. Thus, a 6% rVE was used in the base case scenario. This value, moreover, was similar to the published data of Izurieta et al. 2020.^38^ The main concern about this source is that it compares virosomal-TIV vs. aTIV, and it is used in the CEA as an approximated rVE for SD-TIV vs. aTIV. However, there is no published evidence demonstrating that virosomal-TIV is more effective or more immunogenic than SD-TIV. A sensitivity analysis varying this value between 0% and 6% is provided.

***d.4) Methodological uncertainty***

- While guidelines foster the use of both NHS and societal perspectives^4,16,23^, this evaluation only includes the NHS perspective. However, considering that the target population is normally retired in Spain, the societal perspective seems not to be especially relevant. In accordance with this, time horizon was set at 6 months for direct costs and lifelong for health benefit.
- Although WHO recommends^4,16^ including adverse events in the influenza vaccine modelling, none of the analyzed studies has done so. This is possibly due to the difficulty of estimating adverse events cost and to the general safety of influenza vaccines.

***d.5) Management of uncertainty***

- A scenario analysis is provided testing a broader definition of influenza complication for hospitalizations and 0% rVE for aTIV vs. SD-TIV.
- All upper and lower bound used in the DSA are detailed in the paper, as well as probability distributions used in the PSA, making the sensitivity analysis highly transparent. Sensitivity analyses are presented for both base case and alternative scenario. DSA is presented by tornado diagram, and PSA by acceptability curve, while the cost-effectiveness plane was not represented.

1. ***Ruiz Aragón et al. 2022***

***e.1) Summary of the study***

Authors used a health economic model to assess the costs and outcomes associated with using aQIV or HD-QIV in subjects aged 65+ from the societal and NHS perspectives. Time horizon was 1 year for healthcare costs and life-time for indirect costs and health benefit. Both indirect costs and benefits were discounted at 3% per year. aQIV vs. HD-QIV yielded reductions of 5405 symptomatic cases, 760 primary care visits, 171 emergency room visits, 442 hospitalizations, and 26 deaths in Spain each year. Life-years (LYs) and quality-adjusted LYs (QALYs) increases by 260 and 206, respectively, each year. Savings from a direct medical payer perspective are EUR 63.6 million, driven by the lower aQIV vaccine price and a minor advantage in effectiveness. From a societal perspective, savings increase to EUR 64.2 million. When vaccine prices are assumed equal, aQIV remains dominant compared to HD-QIV. Potential savings are estimated at over EUR 61 million in vaccine costs alone. Therefore, authors affirm that aQIV provides a highly cost-effective alternative to HD-QIV for people aged 65+ in Spain.

***e.2) Model uncertainty***

- No relevant uncertainties were detected. The model type was adequately chosen.

***e.3) Parametric uncertainty***

- Although the authors indicate that QIV absolute effectiveness has been used as the basis for VE, neither the value itself nor its source are mentioned. This is important, as the effect of rVE might be greater or lower based on the absolute VE set in the model.
- rVE for HD-QIV vs. SD-QIV was estimated on the basis of DiazGranados 2014,^42^ assuming equivalence in the rVE between the quadrivalent and trivalent formulation. Thus HD-QIV VE was estimated by applying this rVE on the basal VE of QIV. aQIV VE was estimated through an *ad-hoc* meta-analysis that estimated the rVE of aQIV vs. HD-QIV. However, this meta-analysis raises a few methodological doubts. First, no systematic review was conducted. The list of studies used derives from a previous meta-analysis (Coleman et al. 2021^47^), but only some of them were used in this CEA, and the inclusion and exclusion criteria are not declared in the report. These were integrated with a literature search, which was not systematic, including studies published after Coleman et al. 2021. As a result, study selection bias may be present. Besides, the meta-analysis was affected by high heterogeneity, and the results were not statistically significant. Based on this, authors affirm that there is no difference of efficacy between aQIV and HD-QIV. This yields that the ICER is basically determined by the difference in vaccine price, being aQIV price cheaper than HD-QIV. However, with high heterogeneity, that has not been explored to determine causes, results should be considered inconclusive.
- While influenza-related disutilities have been derived from the same Spanish study as almost all other CEA here considered, the age groups of this model differ from those reported in the original disutility study,^39^ however, the way the original disutilities have been mapped on the model age groups is not explained.

***e.4) Methodological uncertainty***

- Burden of the disease is estimated on the basis of just three seasons, while WHO guidelines^4,16^ indicate that it should be based on a minimum of five to buffer season variability.
- Although WHO recommends^4,16^ including adverse events in the influenza vaccine modelling, none of the analyzed studies has done so. This is possibly due to the difficulty of estimating adverse events cost and to the general safety of influenza vaccines.

***e.5) Management of uncertainty***

- To manage uncertainty derived from effectiveness data, this study includes an alternative scenario with aQIV vs. HD-QIV rVE based on the original meta-analysis by Coleman et al. 2021.^47^ The studies included in that meta-analysis were all observational studies affected by moderate risk of bias. Influenza outcomes are mixed (inpatient, emergency department, and general practice), and the definition of influenza cases is not reported. Heterogeneity of the meta-analysis is high and results not significant (*I^2^*=94.5%; p<0.01), thus the reliability of the meta-analysis is limited. Moreover, the difference between the two rVE values used in the base case and alternative scenario is very small; thus, this analysis does not seem to have an impact on results. In exchange, when just a rVE 0.05% is given in favor of the comparator (lower bound scenario), it becomes cost-effective at a 25,000€/QALY threshold. This result is not discussed.
- Both PSA and DSA have been carried out; however, DSA input data and probability distributions used in the PSA have not been reported.

1. ***Fochesato et al. 2022***

***f.1) Summary of the study***

This study evaluated the cost-effectiveness of aQIV vs. egg-based standard-dose QIV (QIVe) in the elderly population, from the payer and societal perspective in Spain. A dynamic transmission model, which accounts for herd protection, was used to predict the number of medically attended infections in Spain. A decision tree structure was used to forecast influenza-related costs and benefits. Influenza-related probabilities of outpatient visit, hospitalization, work absenteeism, mortality, and associated utilities and costs were extracted from Spanish and European published literature. Relative vaccine effectiveness (rVE) of aQIV vs. QIVe was sourced from two different meta-analyses: the first meta-analysis was informed by laboratory-confirmed influenza studies only, resulting in a rVE = 34.6% (CI95% 2–66%) in favor of aQIV; the second meta-analysis included real world evidence influenza-related medical encounters outcomes, resulting in a rVE = 13.9% (CI95% 4.2–23.5%) in benefit of aQIV. All costs were expressed in 2021 euros. Results indicate that replacing QIVe with aQIV in the Spanish elderly population would prevent on average 43,664 influenza complicated cases, 1111 hospitalizations, and 569 deaths (with a rVE = 34.6%) or 19,104 influenza complicated cases, 486 hospitalizations, and 252 deaths (with a rVE = 13.9%). When the rVE of aQIV vs. QIVe is 34.6%, the incremental cost per quality adjusted life years (QALY) gained was €2240 from the payer; from the societal perspective, aQIV was cost saving compared with QIVe. If the rVE was 13.9%, the incremental cost per QALY was €6694 and €3936 from the payer and societal perspective, respectively. Sensitivity analyses validated the robustness of these findings. Results indicate that replacing QIVe with aQIV in the Spanish elderly population is a cost-effective strategy for the Spanish healthcare system.

***f.2) Model uncertainty***

- No relevant uncertainties were detected. The model type was adequately chosen.

***f.3) Parametric uncertainty***

- Contact maps and attack rate were derived from Italian data. Altogether, 9 seasons were considered for the modelling. Apparently, data were considered transferable by authors when at least one of the viral strains was circulating in both Italy and Spain in one season. However, when comparing circulating strains, we have seen that often the coinciding strain is H1N1 against which all vaccines have high effectiveness in the elderly, while in four seasons either H3N2 or B (or both) were present in one of the two countries and absent in the other.
- QIVe absolute VE was extracted from a model carried out for the Italian HTA of Fluad Tetra;^48^ however, the primary source was the meta-analysis from Belongia et al. 2016 ^30^, and a meta-regression of immunogenicity data ^49^. We could not trace the resulting data in none of the cited sources. Belongia et al. 2021 is a systematic review and meta-analysis of 56 observational studies, including general population attending primary care for influenza-like symptoms. Test-negative design with outpatient recruitment based on predefined criteria and influenza confirmation by PCR were the most relevant selection criteria for inclusion of the studies. This may represent a limitation; in fact, other appropriate study designs (e.g., prospective or retrospective noninterventional cohort and case-control studies) are not included. This source does not provide quality assessment of the included studies, and there is no adjustment of confounding factors, no information on the method of exposure assessment, and finally, there is no analysis of the risk of misclassification.
- rVE for aQIV vs. QIVe was derived from the meta-analysis carried out in the very same Health Technology Assesment^48^ for aTIV vs. eTIV, assuming relative effectiveness equivalence between the respective tri- and quadri-valent formulations. Not enough information on this meta-analysis was available in the HTA publication to assess the quality of the results.
- In general, an extensive use of Italian data has been done, even when Spanish data were available. This was the case with utility data, where author used an observational study carried out in Italy, while an appropriate study^39^ was available for Spain, which was indeed used in all other available CEAs.^11-14,17,18^ Furthermore, discrepancies were detected in the disutility values with respect to the cited source.

***f.4) Methodological uncertainty***

- Among all available vaccine indicated for the model population in Spain, authors chose comparing aQIV to QIVe. Other relevant comparators, such as HD-QIV have not been considered alleging that, in the elderly population in Spain, aQIV had already demonstrated to be cost-saving compared with HD-QIV^13^. However, the study by Redondo et al. 2021^11^ was already available and also cited in this CEE, and its conclusion was that HD-QIV is cost-effective compared to aQIV from the NHS perspective. This evidence discrepancy is not discussed by Fochesato et al., but HD-QIV was not included as a comparator, nor were other potential comparators such as QIV, QIVc or QIVr that are current alternatives in the Spanish market for this population.
- In dynamic transition models calibration is crucial to secure that the epidemiological model is appropriately modeling the influenza season, but it was not reported. Furthermore, epidemiologic data are transferred from Italian sources.
- Although WHO recommends^4,16^ including adverse events in the influenza vaccine modelling, none of the analyzed studies has done so. This is possibly due to the difficulty of estimating adverse events cost and to the general safety of influenza vaccines.

***f.5) Management of uncertainty***

- An alternative scenario was estimated with rVE coming from Coleman et al. 2021. ^47^ As for Coleman et al. 2021, it is a meta-analysis of observational studies with various designs, RCTs were only admitted with a cluster design. The studies included in Coleman et al. 2021 were affected by moderate risk of bias. Flu outcomes are mixed (inpatient, emergency department, and general practice). The definition of influenza cases is not informed. Heterogeneity of the meta-analysis is high and results non-significant (*I^2^*=94,5%; p<0,01), included studies show very different results, thus the reliability of the meta-analysis is limited. A thorough analysis of the possible biases affecting this meta-analysis has been previously published.^50^

1. ***Ruiz Aragón et al. 2023***

***g.1) Summary of the study***

Authors used a health economics model to evaluate the cost effectiveness in people ≥65 years old in Spain of a recently licensed recombinant quadrivalent influenza vaccine (QIVr), which contains three times the standard dose of influenza virus hemagglutinin but no neuraminidase, compared with an MF59-adjuvanted quadrivalent influenza vaccine (aQIV). Based on current tender prices in Spain and an assumption that QIVr is 10.7% relatively more effective than aQIV, the incremental cost-effectiveness ratio (ICER) for QIVr was EUR 101,612.41 per quality-adjusted life year (QALY) gained. To meet the Spanish willingness-to-pay threshold of EUR 25,000 per QALY gained, QIVr would need to be 34.1% relatively more effective than aQIV. In a probabilistic sensitivity analysis conducted to confirm the robustness of the analysis, 99.7% of simulations for QIVr were higher than the willingness-to-pay curve. Authors suggest that QIVr is not currently a cost-effective influenza vaccine option relative to aQIV for older persons living in Spain.

***g.2) Model uncertainty***

- No relevant uncertainties were detected. The model type was adequately chosen.

***g.3) Parametric uncertainty***

- Vaccine effectiveness only includes rVE, which is based on a unique single-season retrospective observational study comparing QIVr to the adjuvanted trivalent influenza vaccine aTIV, in older adults aged ≥65 years in the Medicare fee-for-service database during the 2019–2020 season^51^. Study data were truncated on February 2020 to avoid overlapping with the raising Covid-19 pandemics. This study found that the rVE of QIVr vs. aTIV was 5.6% (95% CI, −0.6 to 11.4) for the prevention of influenza-related hospital encounters and 10.7% (95% CI, 2.7–17.9%) for the prevention of influenza-related inpatient stays. This latter was the rVE used in the modelling and applied to all outcomes.

***g.4) Methodological uncertainty***

- This study is based on a previously developed static decision-tree that was used both in Ruiz Aragón et al. 2020^18^ and 2021^13^. In the paper, the perspective of the analysis is not declared; however, considering that productivity loss costs are included, we understand that it assumes the societal perspective. Nonetheless, disaggregated results are not reported, and NHS perspective is not presented as separated, while applicable guidelines^4,16^ clearly recommend including detailed results.
- Burden of the disease is estimated from the data of the Spanish Influenza Surveillance System on the basis of just three seasons, while WHO guidelines^4,16^ indicate that it should be based on a minimum of five to buffer season variability.
- Although WHO recommends^4,16^ including adverse events in the influenza vaccine modelling, none of the analyzed studies has done so. This is possibly due to the difficulty of estimating adverse events cost and to the general safety of influenza vaccines.

***g.5) Management of uncertainty***

- DSA and PSA were carried out to test the robustness of the model, which are both represented in the paper with a tornado plot and the cost-effectiveness plane, respectively. While DSA upper and lower bounds were presented, their source is not cited. Probability distribution for PSA is not presented either. Considering the uncertainty of the available effectiveness data, authors carried out a scenario analysis in which they fixed the ICER at 25,000€/QALY, which is commonly considered as the acceptability threshold in Spain, and calculated back the necessary rVE for QIVr vs. aQIV, it resulted in 34.12%, authors discuss that this efficacy has not been reached even in the RCT.
