# Supplementary material for: Critical assessment of uncertainty in economic evaluations on influenza vaccines for the elderly population in Spain
Source: BMC Infect Dis. 2025 Feb 1;25:152. doi: 10.1186/s12879-025-10442-3 (PMC11786407; doi:10.1186/s12879-025-10442-3)
Supplement: Supplementary file 5 — Supplementary Material 5. [file 12879_2025_10442_MOESM5_ESM.pdf]

Transparent Uncertainty ASsessmenT (TRUST) tool v1.0

Please use the drop-down lists to fill in this framework. Explanatory notes and examples are provided on the 'Definitions' sheet. Use the 'Remarks' column (M) to provide detail on responses.

DISCLAIMER: When in doubt over whether something is uncertain or not, please select Yes or Intransparent! When in doubt over where to record an uncertain aspect, follow your own judgement, even if it means recording it multiple times!

TRUST Tool

TRUST Definitions

TRUST Summary

Remove contents

Ruiz Aragón et al. 2022

		Sources of uncertainty					Impact on cost effectiveness			
	**Item**	**Lack of transparency:** Lack of clarity in presentation, description, justification? *Please select Yes / No / NA*	**Methods:** Violation of best research practices / existing guidelines/ reference case? *Please select Yes / No / NA / Intransparent*	**Imprecision:** Particularly wide CIs, very small sample size? *Please select Yes / No / NA / Intransparent*	**Bias:** Confounding, risk of bias, or indirectness? *Please select Yes / No / NA / Intransparent*	**Unavailability:** Lack of data, insight? *Please select Yes / No / NA / Intransparent*	**Probabilistic sensitivity analysis:** The identified uncertainty is NOT fully reflected in the PSA? Confirm: *Please select Yes / No / NA*	**Scenario analysis:** The identified uncertainty is NOT explored in scenario analysis? Confirm: *Please select Yes / No / NA*	**Does this uncertainty have an impact on cost effectiveness (given PSA, scenarios, or judgement)?** *Please select Likely high / Likely low / Likely no impact / Unknown*	**Remarks**
**Context / scope**	*PICOTP (Patients, Intervention, Comparators, Outcomes, Time, Perspective)*	*No*	*No*	*Not applicable*	*No*	*No*	*Not applicable*	*No*	*Likely no impact*	The defition of PICOT is adequate to the objective of the study. Epidemiology has been derived from adequate Spanish sources.
**Model structure**	*Health states and how they relate to each other*	*No*	*No*	*Not applicable*	*No*	*Not applicable*	*Yes*	*No*	*Likely no impact*	The structure of the model is adequate to the objective of the study
**Selection of evidence**	*Identification and selection of sources for evidence on effectiveness, safety, utilities & costs*	*Yes*	*Yes*	*Not applicable*	*Yes*	*No*	*Not applicable*	*Yes*	*Likely high*	Various potential sources of bias have been identified and they have not been fully addressed in the OWSA
**Model – Inputs**	*Transition probabilities / time to event / accuracy estimates*	*No*	*No*	*No*	*Yes*	*Intransparent*	*Yes*	*Yes*	*Likely high*	Data from three seasons were considered. However, probability of event data were extracted from another model, which do not cite the primary source of the used data.
**Model – Inputs**	*Relative effectiveness estimate*	*Yes*	*Yes*	*Yes*	*Yes*	*No*	*Yes*	*No*	*Likely high*	based on a home-made metaanalysis not based on systematic review, result was
**Model – Inputs**	*Adverse events*	*No*	*Yes*	*NA*	*NA*	*Yes*	*NA*	*NA*	*Unknown*	AE were not accounted for
**Model – Inputs**	*Utilities*	*Yes*	*Intransparent*	*No*	*Yes*	*No*	*Yes*	*Yes*	*Unknown*	
**Model – Inputs**	*Resource use & costs*	*No*	*No*	*Yes*	*No*	*No*	*Yes*	*Yes*	*Likely high*	Healthcare costs are selected in a partial way.
**Model – Implementation**	*Technical implementation*	*No*	*No*	*Not applicable*	*Not applicable*	*Not applicable*	*Not applicable*	*Not applicable*	*Not applicable*	
**Outcomes**	*ICER, costs, life-years, QALYs gained*	*No*	*Not applicable*	*Not applicable*	*Not applicable*	*Not applicable*	*Not applicable*	*Not applicable*	*Not applicable*	

Key: CI - credible interval; EVPI - Expected value of perfect information; NA - Not applicable; PICOTP - Population, Intervention, Comparison, Outcomes, Time, Perspective; PSA - probabilistic sensitivity analysis
