# Supplementary material for: Critical assessment of uncertainty in economic evaluations on influenza vaccines for the elderly population in Spain
Source: BMC Infect Dis. 2025 Feb 1;25:152. doi: 10.1186/s12879-025-10442-3 (PMC11786407; doi:10.1186/s12879-025-10442-3)
Supplement: Supplementary file 6 — Supplementary Material 6. [file 12879_2025_10442_MOESM6_ESM.pdf]

Transparent Uncertainty ASsessmenT (TRUST) tool v1.0

Please use the drop-down lists to fill in this framework. Explanatory notes and examples are provided on the 'Definitions' sheet. Use the 'Remarks' column (M) to provide detail on responses.

DISCLAIMER: When in doubt over whether something is uncertain or not, please select Yes or Intransparent! When in doubt over where to record an uncertain aspect, follow your own judgement, even if it means recording it multiple times!

TRUST Tool

TRUST Definitions

TRUST Summary

Remove contents

Ruiz-Aragón et al. 2020

			Sources of uncertainty					Impact on cost effectiveness			
		Item	**Lack of transparency:** Lack of clarity in presentation, description, justification? *Please select Yes / No / NA*	**Methods:** Violation of best research practices / existing guidelines/ reference case? *Please select Yes / No / NA / Intransparent*	**Imprecision:** Particularly wide CIs, very small sample size? *Please select Yes / No / NA / Intransparent*	**Bias:** Confounding, risk of bias, or indirectness? *Please select Yes / No / NA / Intransparent*	**Unavailability:** Lack of data, insight? *Please select Yes / No / NA / Intransparent*	**Probabilistic sensitivity analysis:** The identified uncertainty is NOT fully reflected in the PSA? Confirm: *Please select Yes / No / NA*	**Scenario analysis:** The identified uncertainty is NOT explored in scenario analysis? Confirm: *Please select Yes / No / NA*	**Does this uncertainty have an impact on cost effectiveness (given PSA, scenarios, or judgement)?** *Please select Likely high / Likely low / Likely no impact / Unknown*	**Remarks**
**Context / scope**		*PICOTP (Patients, Intervention, Comparators, Outcomes, Time, Perspective)*	*Yes*	*No*	*Not applicable*	*No*	*No*	*Not applicable*	*Yes*	*Likely no impact*	The defition of PICOT is adequate to the objective of the study; however, the subselection of the study population is not clearly justified. Epidemiology has been derived from adequate Spanish sources.
**Model structure**		*Health states and how they relate to each other*	*Yes*	*No*	*Not applicable*	*No*	*Not applicable*	*Yes*	*No*	*Likely no impact*	The structure of the model is adequate to the objective of the study
**Selection of evidence**		*Identification and selection of sources for evidence on effectiveness, safety, utilities & costs*	*Yes*	*Yes*	*Not applicable*	*Yes*	*No*	*Not applicable*	*Yes*	*Likely high*	It seems not that input were always carefully chosen at the best of available evidence
**Model**	**Inputs**	*Transition probabilities / time to event / accuracy estimates*	*Yes*	*No*	*No*	*Yes*	*Intransparent*	*Yes*	*Yes*	*Likely low*	Probabilities are based on 3 influenza seasons
		effectiveness estimate	*Yes*	*No*	*No*	*Yes*	*Yes*	*Yes*	*Yes*	*Unknown*	
		Adverse events	*No*	*Yes*	*NA*	*NA*	*Yes*	*NA*	*NA*	*Unknown*	Adverse events were not accounted for
		Utilities	*Yes*	*Intransparent*	*No*	*Yes*	*Yes*	*Yes*	*Yes*	*Unknown*	
		Resource use & costs	*No*	*No*	*Yes*	*Yes*	*No*	*Yes*	*Yes*	*Likely high*	Healthcare costs have been arbitrarily extracted from official BOEs
	**Implementation**	*Technical implementation*	*No*	*Intransparent*	*Not applicable*	*Not applicable*	*Not applicable*	*Not applicable*	*Not applicable*	*Not applicable*	
**Outcomes**		*ICER, costs, life-years, QALYs gained*	*No*	*Not applicable*	*Not applicable*	*Not applicable*	*Not applicable*	*Not applicable*	*Not applicable*	*Not applicable*	

Key: CI - credible interval; EVPI - Expected value of perfect information; NA - Not applicable; PICOTP - Population, Intervention, Comparison, Outcomes, Time, Perspective; PSA - probabilistic sensitivity analysis
