# Supplementary material for: Critical assessment of uncertainty in economic evaluations on influenza vaccines for the elderly population in Spain
Source: BMC Infect Dis. 2025 Feb 1;25:152. doi: 10.1186/s12879-025-10442-3 (PMC11786407; doi:10.1186/s12879-025-10442-3)
Supplement: Supplementary file 7 — Supplementary Material 7. [file 12879_2025_10442_MOESM7_ESM.pdf]

Transparent Uncertainty ASsessmenT (TRUST) tool v1.0

Please use the drop-down lists to fill in this framework. Explanatory notes and examples are provided on the 'Definitions' sheet. Use the 'Remarks' column (M) to provide detail on responses.

DISCLAIMER: When in doubt over whether something is uncertain or not, please select Yes or Intransparent! When in doubt over where to record an uncertain aspect, follow your own judgement, even if it means recording it multiple times!

TRUST Tool

TRUST Definitions

TRUST Summary

Remove contents

[Title of Appraisal]

		Sources of uncertainty					Impact on cost effectiveness			
	**Item**	**Lack of transparency:** Lack of clarity in presentation, description, justification? *Please select Yes / No / NA*	**Methods:** Violation of best research practices / existing guidelines/ reference case? *Please select Yes / No / NA / Intransparent*	**Imprecision:** Particularly wide CIs, very small sample size? *Please select Yes / No / NA / Intransparent*	**Bias:** Confounding, risk of bias, or indirectness? *Please select Yes / No / NA / Intransparent*	**Unavailability:** Lack of data, insight? *Please select Yes / No / NA / Intransparent*	**Probabilistic sensitivity analysis:** The identified uncertainty is NOT fully reflected in the PSA? Confirm: *Please select Yes / No / NA*	**Scenario analysis:** The identified uncertainty is NOT explored in scenario analysis? Confirm: *Please select Yes / No / NA*	**Does this uncertainty have an impact on cost effectiveness (given PSA, scenarios, or judgement)?** *Please select Likely high / Likely low / Likely no impact / Unknown*	**Remarks**
**Context / scope**	*PICOTP (Patients, Intervention, Comparators, Outcomes, Time, Perspective)*	*No*	*Yes*	*Not applicable*	*No*	*No*	*Not applicable*	*NA*	*Likely no impact*	The evaluation only includes the NHS perspective, while guidelines foster the use of both NHS and societal perspectives.
**Model structure**	*Health states and how they relate to each other*	*Yes*	*No*	*Not applicable*	*No*	*Not applicable*	*No*	*No*	*Likely no impact*	Seem adequate
**Selection of evidence**	*Identification and selection of sources for evidence on effectiveness, safety, utilities & costs*	*No*	*No*	*Not applicable*	*No*	*No*	*Not applicable*	*No*	*Likely low*	Even if there is no RCT evidence for aTIV superiority versus SDTIV it has been assumed that rVE (aTIV versus SD-TIV) = 6%, according to the results of an observational study carried out in Spain [26], is in line with the results of other US observational studies [27,28]. In general, it seems relying on the best evidence available
**Model – Inputs**	*Transition probabilities / time to event / accuracy estimates*	*No*	*No*	*No*	*Yes*	*Yes*	*No*	*No*	*Likely low*	
**Model – Inputs**	*Relative effectiveness estimate*	*No*	*No*	*Yes*	*Yes*	*No*	*No*	*No*	*Likely high*	evidence for aTIV superiority versus SDTIV, it has been assumed that rVE (aTIV versus
**Model – Inputs**	*Adverse events*	*NA*	*Yes*	*NA*	*NA*	*Yes*	*NA*	*NA*	*Unknown*	AE of vaccines were not accounted for
**Model – Inputs**	*Utilities*	*No*	*No*	*No*	*No*	*Yes*	*No*	*Yes*	*Likely low*	Baseline and event (outpatient and inpatient flu) utilities have been taken from two Spanish observational studies, which seem resonable
**Model – Inputs**	*Resource use & costs*	*Yes*	*No*	*Yes*	*No*	*Yes*	*No*	*No*	*Likely high*	Seem adequate
**Model – Implementation**	*Technical implementation*	*No*	*No*	*Not applicable*	*Not applicable*	*Not applicable*	*Not applicable*	*Not applicable*	*Not applicable*	
**Outcomes**	*ICER, costs, life-years, QALYs gained*	*No*	*Not applicable*	*Not applicable*	*Not applicable*	*Not applicable*	*Not applicable*	*Not applicable*	*Not applicable*	

Key: CI - credible interval; EVPI - Expected value of perfect information; NA - Not applicable; PICOTP - Population, Intervention, Comparison, Outcomes, Time, Perspective; PSA - probabilistic sensitivity analysis
