# Supplementary material for: Critical assessment of uncertainty in economic evaluations on influenza vaccines for the elderly population in Spain
Source: BMC Infect Dis. 2025 Feb 1;25:152. doi: 10.1186/s12879-025-10442-3 (PMC11786407; doi:10.1186/s12879-025-10442-3)
Supplement: Supplementary file 8 — Supplementary Material 8. [file 12879_2025_10442_MOESM8_ESM.pdf]

Transparent Uncertainty ASsessmenT (TRUST) tool v1.0

Please use the drop-down lists to fill in this framework. Explanatory notes and examples are provided on the 'Definitions' sheet. Use the 'Remarks' column (M) to provide detail on responses.

DISCLAIMER: When in doubt over whether something is uncertain or not, please select Yes or Intransparent! When in doubt over where to record an uncertain aspect, follow your own judgement, even if it means recording it multiple times!

TRUST Tool | TRUST Definitions | TRUST Summary

Remove contents

Ruiz Aragón et al. 2023

			Sources of uncertainty					Impact on cost effectiveness			
		**Item**	**Lack of transparency:** Lack of clarity in presentation, description, justification? *Please select Yes / No / NA*	**Methods:** Violation of best research practices / existing guidelines/ reference case? *Please select Yes / No / NA / Intransparent*	**Imprecision:** Particularly wide CIs, very small sample size? *Please select Yes / No / NA / Intransparent*	**Bias:** Confounding, risk of bias, or indirectness? *Please select Yes / No / NA / Intransparent*	**Unavailability:** Lack of data, insight? *Please select Yes / No / NA / Intransparent*	**Probabilistic sensitivity analysis:** The identified uncertainty is NOT fully reflected in the PSA? Confirm: *Please select Yes / No / NA*	**Scenario analysis:** The identified uncertainty is NOT explored in scenario analysis? Confirm: *Please select Yes / No / NA*	**Does this uncertainty have an impact on cost effectiveness (given PSA, scenarios, or judgement)?** *Please select Likely high / Likely low / Likely no impact / Unknown*	**Remarks**
**Context / scope**		*PICOTP (Patients, Intervention, Comparators, Outcomes, Time, Perspective)*	*Yes*	*No*	*Not applicable*	*No*	*No*	*Not applicable*	*No*	*Likely no impact*	The defition of PICOT is adequate to the objective of the study. Epidemiology has been derived from adequate Spanish sources, however there is a lack of transparency in the calculation of the population in that it is declared that a 100000 population is used, but afterwards it is said that the population size is based on the data form the National Institute of Statistics.
**Model structure**		*Health states and how they relate to each other*	*No*	*No*	*Not applicable*	*No*	*Not applicable*	*Yes*	*No*	*Likely no impact*	The structure of the model is adequate to the objective of the study
**Selection of evidence**		*Identification and selection of sources for evidence on effectiveness, safety, utilities & costs*	*No*	*No*	*Not applicable*	*Yes*	*Yes*	*Not applicable*	*Yes*	*Likely high*	The source used for the rVE is a RW study carried out in a single season. Besides, it is not clear what is the absolute effectiveness and from which source it is derived.
**Model structure**	**Inputs**	*Transition probabilities / time to event / accuracy estimates*	*No*	*Yes*	*No*	*No*	*No*	*Yes*	*Yes*	*Likely high*	Data from just three seasons were considered.
		Relative effectiveness estimate	*No*	*No*	*Yes*	*Yes*	*Yes*	*Yes*	*Yes*	*Likely high*	a RW study carried out in a single season. Besides, it is not clear what is the absolute
		Adverse events	*No*	*Yes*	*NA*	*NA*	*Yes*	*NA*	*NA*	*Unknown*	AE were not accounted for
		Utilities	*Yes*	*Intransparent*	*No*	*Yes*	*No*	*Yes*	*Yes*	*Likely low*	
		Resource use & costs	*No*	*Yes*	*Yes*	*No*	*No*	*Yes*	*Yes*	*Likely high*	The selection of sources of healthcare costs are not justified
	**Implementation**	*Technical implementation*	*No*	*No*	*Not applicable*	*Not applicable*	*Not applicable*	*Not applicable*	*Not applicable*	*Not applicable*	
**Outcomes**		*ICER, costs, life-years, QALYs gained*	*No*	*Not applicable*	*Not applicable*	*Not applicable*	*Not applicable*	*Not applicable*	*Not applicable*	*Not applicable*	

Key: CI - credible interval; EVPI - Expected value of perfect information; NA - Not applicable; PICOTP - Population, Intervention, Comparison, Outcomes, Time, Perspective; PSA - probabilistic sensitivity analysis

References:

ISPOR-SMDM 6; Briggs et al. Med Decis Making 2012;32:722–732
Grimm et al. PharmacoEconomics (2020) 38:205–216
Walker et al. Integrated assessment 4.1 (2003): 5-17.
WHO/IVB/16.05.
GRADE System. Aguayo-Albasini et al. Cir Esp.2014;92(2):82-8
ISPOR-SMDM 7; Eddy et al. Value Health. 2012;15(5):843-850.
