# Supplementary material for: Critical assessment of uncertainty in economic evaluations on influenza vaccines for the elderly population in Spain
Source: BMC Infect Dis. 2025 Feb 1;25:152. doi: 10.1186/s12879-025-10442-3 (PMC11786407; doi:10.1186/s12879-025-10442-3)
Supplement: Supplementary file 9 — Supplementary Material 9. [file 12879_2025_10442_MOESM9_ESM.pdf]

Transparent Uncertainty ASsessmenT (TRUST) tool v1.0

Please use the drop-down lists to fill in this framework. Explanatory notes and examples are provided on the 'Definitions' sheet. Use the 'Remarks' column (M) to provide detail on responses.

DISCLAIMER: When in doubt over whether something is uncertain or not, please select Yes or Intransparent! When in doubt over where to record an uncertain aspect, follow your own judgement, even if it means recording it multiple times!

TRUST Tool

TRUST Definitions

TRUST Summary

Remove contents

Fochesato et al. 2022

		Sources of uncertainty					Impact on cost effectiveness			
	Item	**Lack of transparency:** Lack of clarity in presentation, description, justification? *Please select Yes / No / NA*	**Methods:** Violation of best research practices / existing guidelines/ reference case? *Please select Yes / No / NA / Intransparent*	**Imprecision:** Particularly wide CIs, very small sample size? *Please select Yes / No / NA / Intransparent*	**Bias:** Confounding, risk of bias, or indirectness? *Please select Yes / No / NA / Intransparent*	**Unavailability:** Lack of data, insight? *Please select Yes / No / NA / Intransparent*	**Probabilistic sensitivity analysis:** The identified uncertainty is NOT fully reflected in the PSA? Confirm: *Please select Yes / No / NA*	**Scenario analysis:** The identified uncertainty is NOT explored in scenario analysis? Confirm: *Please select Yes / No / NA*	**Does this uncertainty have an impact on cost effectiveness (given PSA, scenarios, or judgement)?** *Please select Likely high / Likely low / Likely no impact / Unknown*	**Remarks**
**Context / scope**	*PICOTP (Patients, Intervention, Comparators, Outcomes, Time, Perspective)*	*No*	*No*	*Not applicable*	*No*	*NA*	*Not applicable*	*No*	*Likely no impact*	Population is adequately defined based on age distribution from INE and defining the "at risk" population according to flu vaccination recommendations in force at the time of the analysis. Outcomes and perspective are consisten to the objective of the analysis.
**Model structure**	*Health states and how they relate to each other*	*No*	*No*	*Not applicable*	*No*	*Not applicable*	*No*	*No*	*Likely no impact*	The model includes a dynamic module simulating the number of cases in the season, and a decision tree estimating the associated burden in terms of complications and associated cost/QALYs and deaths. Model accounts for herd immunity. Health states are reasonably defined.
**Selection of evidence**	*Identification and selection of sources for evidence on effectiveness, safety, utilities & costs*	*No*	*No*	*Not applicable*	*Yes*	*No*	*Not applicable*	*No*	*Likely low*	The selection of the evidence seem to be the best evidence available, and, when data were not available for Spain, Italian data were used, which seems a good proxy, because the Public Healthcare System are quite similar in both contries.
**Model** – **Inputs**	*Transition probabilities / time to event / accuracy estimates*	*No*	*No*	*No*	*Yes*	*Yes*	*No*	*Yes*	*Unknown*	For the transition module, published contact maps for the Spanish population were used. Influenza generation time, as well as the contact rates of the strains dominant in Spain in the considered seasons were extracted from Italian data, in the absence of published Spanish data. Strains information is based on 9 subsequent flu seasons
**Model** – **Inputs**	*Relative effectiveness estimate*	*No*	*No*	*No*	*Yes*	*No*	*No*	*No*	*Likely high*	adequate; relative effectiveness for aQIV vs. QIV not available; however a reasonable proxy has been used instead.
**Model** – **Inputs**	*Adverse events*	*No*	*Yes*	*NA*	*NA*	*Yes*	*NA*	*NA*	*Unknown*	Adverse events are not accounted for
**Model** – **Inputs**	*Utilities*	*Yes*	*No*	*No*	*No*	*Yes*	*No*	*No*	*Likely high*	mismatches between the cited source and the paper have been detected.
**Model** – **Inputs**	*Resource use & costs*	*No*	*No*	*No*	*Intransparent*	*Yes*	*No*	*No*	*Likely high*	Productivity loss seems correctly defined and sources appropriately selected. How unit healthcare costs have been derived is not clearly described. However, they seem to have little impact on ICER, while ICER seems highly dependent on vaccine cost.
**Model** – **Implementation**	*Technical implementation*	*No*	*No*	*Not applicable*	*Not applicable*	*Not applicable*	*Not applicable*	*Not applicable*	*Not applicable*	
**Outcomes**	*ICER, costs, life-years, QALYs gained*	*No*	*Not applicable*	*Not applicable*	*Not applicable*	*Not applicable*	*Not applicable*	*Not applicable*	*Not applicable*	

Key: CI - credible interval; EVPI - Expected value of perfect information; NA - Not applicable; PICOTP - Population, Intervention, Comparison, Outcomes, Time, Perspective; PSA - probabilistic sensitivity analysis
