# Supplementary material for: Critical assessment of uncertainty in economic evaluations on influenza vaccines for the elderly population in Spain
Source: BMC Infect Dis. 2025 Feb 1;25:152. doi: 10.1186/s12879-025-10442-3 (PMC11786407; doi:10.1186/s12879-025-10442-3)
Supplement: Supplementary file 10 — Supplementary Material 10. [file 12879_2025_10442_MOESM10_ESM.pdf]

Transparent Uncertainty ASsessmenT (TRUST) tool v1.0

Please use the drop-down lists to fill in this framework. Explanatory notes and examples are provided on the 'Definitions' sheet. Use the 'Remarks' column (M) to provide detail on responses.

DISCLAIMER: When in doubt over whether something is uncertain or not, please select Yes or Intransparent! When in doubt over where to record an uncertain aspect, follow your own judgement, even if it means recording it multiple times!

Crépey et al. 2020

TRUST Tool

TRUST Definitions

TRUST Summary

Remove contents

			Sources of uncertainty					Impact on cost effectiveness			
		Item	**Lack of transparency:** Lack of clarity in presentation, description, justification? *Please select Yes / No / NA*	**Methods:** Violation of best research practices / existing guidelines/ reference case? *Please select Yes / No / NA / Intransparent*	**Imprecision:** Particularly wide CIs, very small sample size? *Please select Yes / No / NA / Intransparent*	**Bias:** Confounding, risk of bias, or indirectness? *Please select Yes / No / NA / Intransparent*	**Unavailability:** Lack of data, insight? *Please select Yes / No / NA / Intransparent*	**Probabilistic sensitivity analysis:** The identified uncertainty is NOT fully reflected in the PSA? Confirm: *Please select Yes / No / NA*	**Scenario analysis:** The identified uncertainty is NOT explored in scenario analysis? Confirm: *Please select Yes / No / NA*	**Does this uncertainty have an impact on cost effectiveness (given PSA, scenarios, or judgement)?** *Please select Likely high / Likely low / Likely no impact / Unknown*	**Remarks**
**Context / scope**		*PICOTP (Patients, Intervention, Comparators, Outcomes, Time, Perspective)*	*No*	*No*	*Not applicable*	*No*	*No*	*Not applicable*	*No*	*Likely no impact*	Three different strategies were tested, however, only the first one has been considered as comparing QIV vs. TIV in all eligible population.
**Model structure**		*Health states and how they relate to each other*	*No*	*No*	*Not applicable*	*No*	*Not applicable*	*No*	*No*	*Likely no impact*	Herd immuning considered, and B partial cross-protection considered for TIV in case of B type mismaching
**Selection of evidence**		*Identification and selection of sources for evidence on effectiveness, safety, utilities & costs*	*No*	*Yes*	*Not applicable*	*Yes*	*Yes*	*Not applicable*	*No*	*Unknown*	The source of efficacy data for elderly population is not published, nor its methodology detailed, which may imply a greater or smaller impact according to the design
**Model**	**Inputs**	*Transition probabilities / time to event / accuracy estimates*	*No*	*No*	*No*	*Yes*	*No*	*No*	*No*	*Likely high*	Model parameter choice seem reasonable
		Relative effectiveness estimate	*No*	*Yes*	*No*	*No*	*Yes*	*No*	*Yes*	*Unknown*	elderly population is not published, nor its methodology detailed, which
		Adverse events	*No*	*Yes*	*NA*	*NA*	*Yes*	*NA*	*NA*	*Unknown*	Adverse events were not accounted for
		Utilities	*No*	*No*	*No*	*No*	*No*	*No*	*Yes*	*Likely low*	Selection of data seems adequate
		Resource use & costs			*No*	*No*	*Yes*	*No*	*No*	*Likely high*	Selection of data seems adequate
	**Implementation**	*Technical implementation*	*No*	*No*	*Not applicable*	*Not applicable*	*Not applicable*	*Not applicable*	*Not applicable*	*Not applicable*	
**Outcomes**		*ICER, costs, life-years, QALYs gained*	*No*	*Not applicable*	*Not applicable*	*Not applicable*	*Not applicable*	*Not applicable*	*Not applicable*	*Not applicable*	

Key: CI - credible interval; EVPI - Expected value of perfect information; NA - Not applicable; PICOTP - Population, Intervention, Comparison, Outcomes, Time, Perspective; PSA - probabilistic sensitivity analysis
