# Supplementary material for: Critical assessment of uncertainty in economic evaluations on influenza vaccines for the elderly population in Spain
Source: BMC Infect Dis. 2025 Feb 1;25:152. doi: 10.1186/s12879-025-10442-3 (PMC11786407; doi:10.1186/s12879-025-10442-3)
Supplement: Supplementary file 11 — Supplementary Material 11. [file 12879_2025_10442_MOESM11_ESM.pdf]

Transparent Uncertainty ASsessmenT (TRUST) tool v1.0

Please use the drop-down lists to fill in this framework. Explanatory notes and examples are provided on the 'Definitions' sheet. Use the 'Remarks' column (M) to provide detail on responses.

DISCLAIMER: When in doubt over whether something is uncertain or not, please select Yes or Intransparent! When in doubt over where to record an uncertain aspect, follow your own judgement, even if it means recording it multiple times!

TRUST Tool

TRUST Definitions

TRUST Summary

Remove contents

García et al. 2016

		Sources of uncertainty					Impact on cost effectiveness			
	**Item**	**Lack of transparency:** Lack of clarity in presentation, description, justification? *Please select Yes / No / NA*	**Methods:** Violation of best research practices / existing guidelines/ reference case? *Please select Yes / No / NA / Intransparent*	**Imprecision:** Particularly wide CIs, very small sample size? *Please select Yes / No / NA / Intransparent*	**Bias:** Confounding, risk of bias, or indirectness? *Please select Yes / No / NA / Intransparent*	**Unavailability:** Lack of data, insight? *Please select Yes / No / NA / Intransparent*	**Probabilistic sensitivity analysis:** The identified uncertainty is NOT fully reflected in the PSA? Confirm: *Please select Yes / No / NA*	**Scenario analysis:** The identified uncertainty is NOT explored in scenario analysis? Confirm: *Please select Yes / No / NA*	**Does this uncertainty have an impact on cost effectiveness (given PSA, scenarios, or judgement)?** *Please select Likely high / Likely low / Likely no impact / Unknown*	**Remarks**
**Context / scope**	*PICOTP (Patients, Intervention, Comparators, Outcomes, Time, Perspective)*	*No*	*No*	*Not applicable*	*No*	*No*	*Not applicable*	*No*	*Likely no impact*	Time horizon over lifetime; population "at risk" all ages and "healthy" elderly population. QIV vs. TIV. NHS and societal perspective included
**Model structure**	*Health states and how they relate to each other*	*No*	*No*	*Not applicable*	*No*	*Not applicable*	*No*	*No*	*Likely no impact*	Lifetime static transition model with one-year cycles: A number of events could happen during each cycle, with any subject having a differential probability of the following events: vaccination; suffer influenza infection; seek medical advice for influenza (Primary Care or Emergency Room); suffer influenza-related complications; need hospitalization for complication; death. All survivors from each cycle would begin a new annual cycle.
**Selection of evidence**	*Identification and selection of sources for evidence on effectiveness, safety, utilities & costs*	*No*		*Not applicable*	*Yes*	*No*	*Not applicable*	*Yes*	*Unknown*	
**Model** – **Inputs**	*Transition probabilities / time to event / accuracy estimates*	*No*	*No*	*No*	*Yes*	*No*	*No*	*Yes*	*Unknown*	
**Model** – **Inputs**	*Relative effectiveness estimate*	*Yes*	*No*	*Yes*	*No*	*Yes*	*Yes*	*Yes*	*Unknown*	protection with TIV against B type influenza in case of missmatch, proportional
**Model** – **Inputs**	*Adverse events*	*NA*	*Yes*	*NA*	*NA*	*Yes*	*NA*	*NA*	*Unknown*	
**Model** – **Inputs**	*Utilities*	*No*	*No*	*No*	*No*	*No*	*Yes*	*Yes*	*Likely no impact*	Sources of utilities seem adequate
**Model** – **Inputs**	*Resource use & costs*	*No*	*No*	*No*	*No*	*No*	*Yes*	*Yes*	*Likely low*	Spanish official sources were used and unit costs were averaged when different values were available for the same item.
**Model** – **Implementation**	*Technical implementation*	*No*	*No*	*Not applicable*	*Not applicable*	*Not applicable*	*Not applicable*	*Not applicable*	*Not applicable*	
**Outcomes**	*ICER, costs, life-years, QALYs gained*	*No*	*Not applicable*	*Not applicable*	*Not applicable*	*Not applicable*	*Not applicable*	*Not applicable*	*Not applicable*	Costs and benefits discounted at the adequate rate for Spain

Key: CI - credible interval; EVPI - Expected value of perfect information; NA - Not applicable; PICOTP - Population, Intervention, Comparison, Outcomes, Time, Perspective; PSA - probabilistic sensitivity analysis
